# Supplementary material for: 3D N-heterocyclic covalent organic frameworks for urea photosynthesis from NH3 and CO2
Source: Nat Commun. 2025 Jan 28;16:1106. doi: 10.1038/s41467-025-56307-w (PMC11775333; doi:10.1038/s41467-025-56307-w)
Supplement: Supplementary file 1 — Supplementary Information [file 41467_2025_56307_MOESM1_ESM.pdf]

Supplementary Materials

3D N-heterocyclic Covalent Organic Frameworks for Urea Photosynthesis from NH_3 and CO_2

Ning Li,[1] Jiale Zhang,[1] Xiangdong Xie,[2] Kang Wang,*[1] Dongdong Qi,*[1] Jiang Liu,[2] Ya-Qian Lan,[2] and Jianzhuang Jiang*[1]

[1] Beijing Key Laboratory for Science and Application of Functional Molecular and Crystalline Materials, Department of Chemistry and Chemical Engineering, School of Chemistry and Biological Engineering, University of Science and Technology Beijing, Beijing 100083, China.

[2] Guangdong Provincial Key Laboratory of Carbon Dioxide Resource Utilization, School of Chemistry, South China Normal University, Guangzhou, 510006, China

* Corresponding author Email: kangwang@ustb.edu.cn (K.W.); qdd@ustb.edu.cn (D.Q.); jianzhuang@ustb.edu.cn (J.J.).

Experimental Section

Chemicals

All the starting materials were purchased from TCI or Admas, and used without further purification. All solvents were dried and distilled according to conventional methods.

Synthesis of 5,5'-(pyrazine-2,5-diyl) diisophthalaldehyde (PDDP)

2,5-dibromopyrazine (0.5 g, 2.1 mmol), 3, 5- diformylphenyl boronic acid (1.5 g, 5.8 mmol), K_2CO_3 (1.4 g, 10 mmol), and $Pd(PPh_3)_4$ (0.12 g, 0.1 mmol) in 120 mL of a mixed solvent of THF and water (v/v, 3:1) were added to a 250 mL round-bottomed flask equipped with a magnetic stirrer bar. A condenser was attached to the flask and the mixture was degassed by bubbling N_2 for 30 min. The reaction mixture was heated at 95 °C for 30 hours and then cooled to room temperature. The aqueous layer was removed by pipette. THF was removed under reduced pressure and the residue was dissolved in CH_2Cl_2 (200 mL). The solution was washed with saturated brine solution and water and dried with anhydrous Na_2SO_4. The volatiles were removed under reduced pressure and the residue was purified by EtOAc and CH_2Cl_2 to give the PDDP as a powder in a yield of 80%.[S1]

Synthesis of 5,5'-(1,2,4,5-tetrazine-3,6-diyl) bis-benzene-1,3-diamine (TBBD)

5-ethynylbenzene-1,3-diamine (5 g, 0.037 mol) is dissolved in 60 mL of a mixed solvent of ethanol and hydrazine hydrate (v/v, 2:1). Then sulphur powder (2.5 g, 0.078 mol) were added into the mixture. The resulting solution was heated at 90 °C under vigorous stirring for 72 h. A bright golden-yellow colored thick suspension was observed which was filtered and washed with ethanol and acetone several times. The product was then dried at 60 °C in a vacuum oven overnight to give the TBBD as a powder with a yield of 10%.[S2]

Characterization

^{13}C solid-state nuclear magnetic resonance (^{13}C ssNMR) spectra were recorded on a 400 MHz WB Solid-State NMR Spectrometer (Bruker AVANCE III). Fourier transform infrared

(FT-IR) spectra were recorded by using a Bruker Tensor 37 spectrometer. Thermogravimetric analysis (TGA) was recorded on a NETZSCH STA 449F3 thermal analyzer under N_2. Powder X-ray diffraction (PXRD) patterns were acquired on a Shimadzu XRD-6000 diffractometer using Cu-Kα radiation (I = 1.54056 Å) at room temperature. The sorption isotherm for N_2 and CO_2 were measured by using an ASAP 2020 Plus with ultra-high-purity gas. The sorption isotherm for NH_3 was measured by using a BSD-PMC. Scanning electron microscopy (SEM) images were recorded on a Gemini SEM 300 scanning electron microscope. High-Resolution Transmission Electron Microscope (HR-TEM) and energy dispersive spectroscopy (EDS) mapping images were taken on a JEM-ARM200F electron microscope operated at 200 kV. X-ray photoelectron spectroscopy (XPS) measurements were carried out on Thermo Scientific K-Alpha. UV-vis diffuse reflectance absorption spectra (DRS) were recorded on a Shimadzu UV-2600 spectrophotometer with $BaSO_4$ as the reference. Photoelectrochemical experiments (i.e., Mott-Schottky analysis, impedance measurement, and transient photocurrent measurement) were carried out on the CHI760E workstation (CHI Instruments, USA). High-resolution mass spectra (HR-MS) were recorded on a Bruker Solarix.

***In-situ* Diffuse Reflectance Infrared Fourier Transform (DRIFT) measurement.**

In-situ DRIFT measurements were performed on BRUKER Vertex 70V. In the instrument, a cell containing two ZnSe windows and one quartz window was placed inside the Praying Mentis chamber. The photocatalyst was flattened and compacted on the sample stage. After 1 h drying at room temperature, the glass slide was placed into the center and the cell was then closed. Subsequently, pure CO_2 (99.99%) and NH_3 (99.99%) were passed into the reactor. As the adsorption time increased, the *in situ* IR signals of the adsorption were collected every 60 s with an MCT detector. In addition, the pure CO_2 (99.99%) and NH_3 (99.99%) were passed into the reactor, and the sample was irradiated by visible light and in-situ IR signals were collected by MCT detector after each 1 minute of the reaction.

CO_2 and NH_3 sorption measurements.

Activation of 3D-TPT-COF, 3D-PDDP-COF, and 3D-TBBD-COF were performed under a

dynamic vacuum for 1 h at 50 ºC (2 ºC min^{-1}) and then a dynamic vacuum for 12 h at 120 ºC (2 ºC min^{-1}). CO_2 and NH_3 sorption measurements were performed on the activated samples by using ASAP 2020 Plus and BSD-PM. All gases used for the adsorption measurements were high-purity grade (99.999%).

Photocatalytic synthesis of urea.

The photocatalytic experiment is completed in a self-assembled photocatalytic reaction platform. Specifically, 5 mg of photocatalyst was dispersed in 15 mL of deionized water, and then the mixed solution was sealed in a glass reactor with a quartz plate cover. NH_3 and CO_2 (v/v, 2:1) were passed into another glass reactor containing 15 mL of deionized water, which was then added to the first glass reactor. The mixture was darkened with continuous stirring and then irradiated continuously for 2 hours by using a 300W Xe lamp as a light source (light intensity of 97 mW cm^{-2}). The concentration of urea in the supernatant was determined by the diacetylmonoxime method. After catalysis, 3D-TBBD-COF was separated from the mixture at the end of the reaction by centrifugation. The solid was washed and subsequently reused for the next cycle. The same procedure was repeated for five cycles to assess the recyclability of this catalyst.

Supplementary note.

To evaluate the possible viability of urea production from CO_2 and NH_3, a preliminary techno-economic analysis was carried out to calculate the costs of electrochemical urea production and photocatalytic urea production, which are then compared with the market price of industrial urea, USD0.41/kg. Only the costs of energy and feedstock input were calculated without other costs associated with practical production or infrastructure.[S3-S5].

Electrocatalytic urea production cost

Operation condition: 40 mA cm^{-2} (average cell voltage: 2.7 V); Faradaic efficiency: 11.26%; Production rate: 15.13 mmol g^{-1} h^{-1} (0.91 mg h^{-1})

$$m_{urea}=0.91\ \text{mg h}^{-1}\times 1\ \text{KWh}\div(2.7\ \text{V}\times 0.04\ \text{A})=8.425\ \text{g}$$

Thus 8.425 g urea costs 1 kWh electricity. The production of 1 kg urea will cost 118.69 kWh of electricity. According to the total reaction listed below:

$$2NH_3+CO_2=CO(NH_2)_2+H_2O$$

1 kg urea consumes 733.1 g CO_2 and 499.5 g NH_3, where the industrial CO_2 price is USD 0.03/kg and the price of industrial NH_3 is USD 0.10/kg. Therefore, the CO_2 cost is USD 0.022 per kilogram of urea, and the NH_3 cost is USD 0.049 per kilogram of urea. Assuming the price of renewable electricity is USD3/kWh. Besides, the price of DI water is estimated to be USD0.008/kg. Thus, we can roughly estimate a urea production cost of *ca.* USD3.60/kg-urea.

Photocatalytic urea production cost

According to the total reaction listed below:

$$2NH_3+CO_2=CO(NH_2)_2+H_2O$$

1 kg urea consumes 733.1 g CO_2 and 499.5 g NH_3, where the industrial CO_2 price is USD 0.03/kg and the price of industrial NH_3 is USD 0.10/kg. Therefore, the CO_2 cost is USD 0.022 per kilogram of urea, and the NH_3 cost is USD 0.049 per kilogram of urea. Besides, the price of DI water is estimated to be USD 0.008/kg. Thus, the production cost of urea can be roughly estimated at *ca.* 0.08/kg-urea. Notably, most commercial urea plants manufacture in a centralized model with a production scale of 800 to 3500 metric tons per day, significantly increasing storage and transport costs. Nearly half of the annually produced energy is wasted during the transportation process because of inefficient energy storage/conversion systems.

Calculation Methods and Additional explanation to the different adsorbing behaviors of CO_2-Tz and NH_4^+-Tz.

All the calculation in this work is carried out at the level of M06-2X/6-311G(d)[S6] using Gaussian 16 B.01 Software[S7] and Multiwfn 3.8.[S8,S9] The polarizable continuum model using the integral equation formalism variant (IEFPCM) solvent mode[S10] was adopted in the whole calculation. In addition, the different adsorbing behaviors of CO_2-Tz and NH_4^+-Tz must be clarified. When CO_2 is absorbed onto the N atom in the Tz moiety, the attracting mechanism

is a typical chemical absorption with an energy barrier of 0.26 eV, which must be overcome in the forming process of CO_2-Tz. However, as a typical physical absorption, the NH_4^+-Tz could be directly constructed with no energy barrier. Although the binding energy of NH_4^+-Tz (13 kJ mol^{-1}) is much lower than that of CO_2-Tz (85 kJ mol^{-1}), the formation rate of NH_4^+-Tz is not slower than that of CO_2-Tz.

Results and Discussion

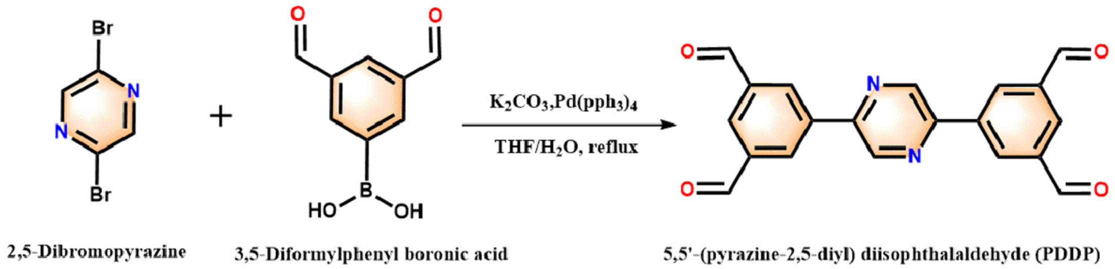

Supplementary Fig. 1| Synthesis of PDDP. The reaction of 2,5-dibromopyrazine with 3, 5-diformylphenyl boronic acid in THF and water (v/v, 3:1) generates PDDP.

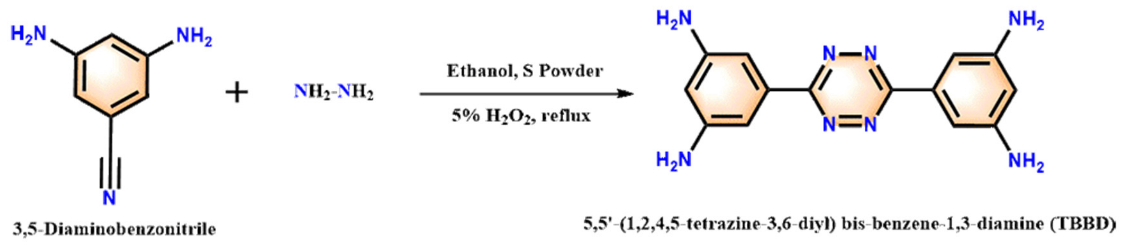

Supplementary Fig. 2| Synthesis of TBBD. The reaction of 5-ethynylbenzene-1,3-diamine with sulphur powder in ethanol and hydrazine hydrate (v/v, 2:1) generates TBBD.

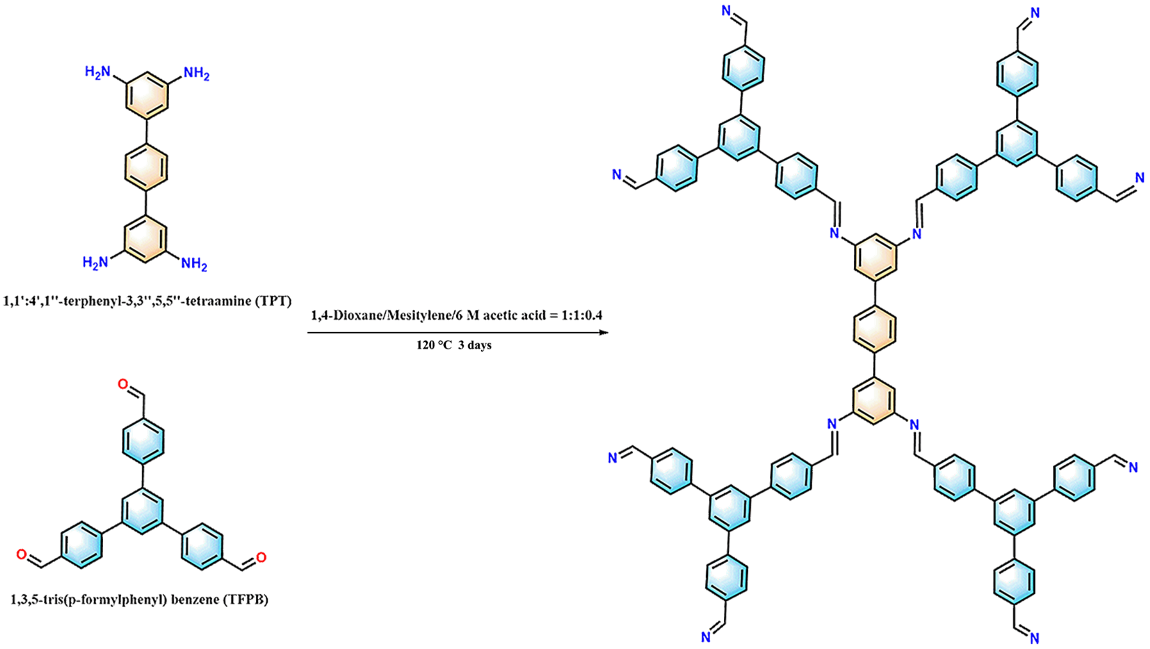

Supplementary Fig. 3 | Synthesis of 3D-TPT-COF. The solvothermal reaction of TFPB with TPT in 1,4-dioxnae, mesitylene, and 6 M acetic acid generates 3D-TPT-COF.

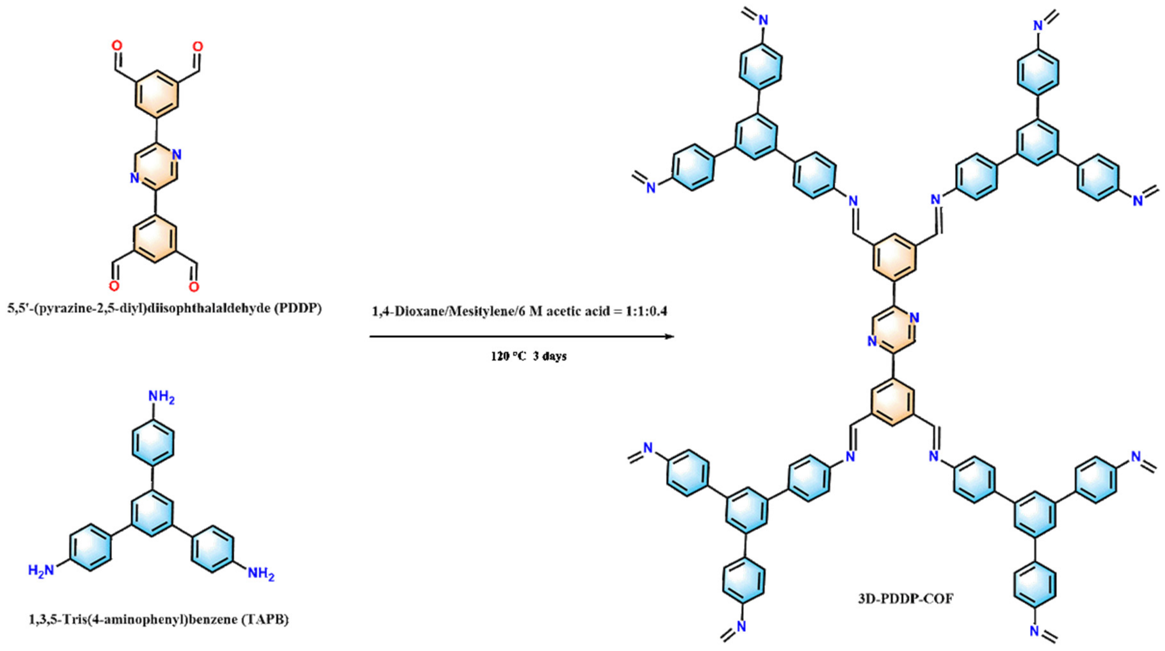

Supplementary Fig. 4 | Synthesis of 3D-PDDP-COF. The solvothermal reaction of TAPB with PDDP in 1,4-dioxnae, mesitylene, and 6 M acetic acid generates 3D-PDDP-COF.

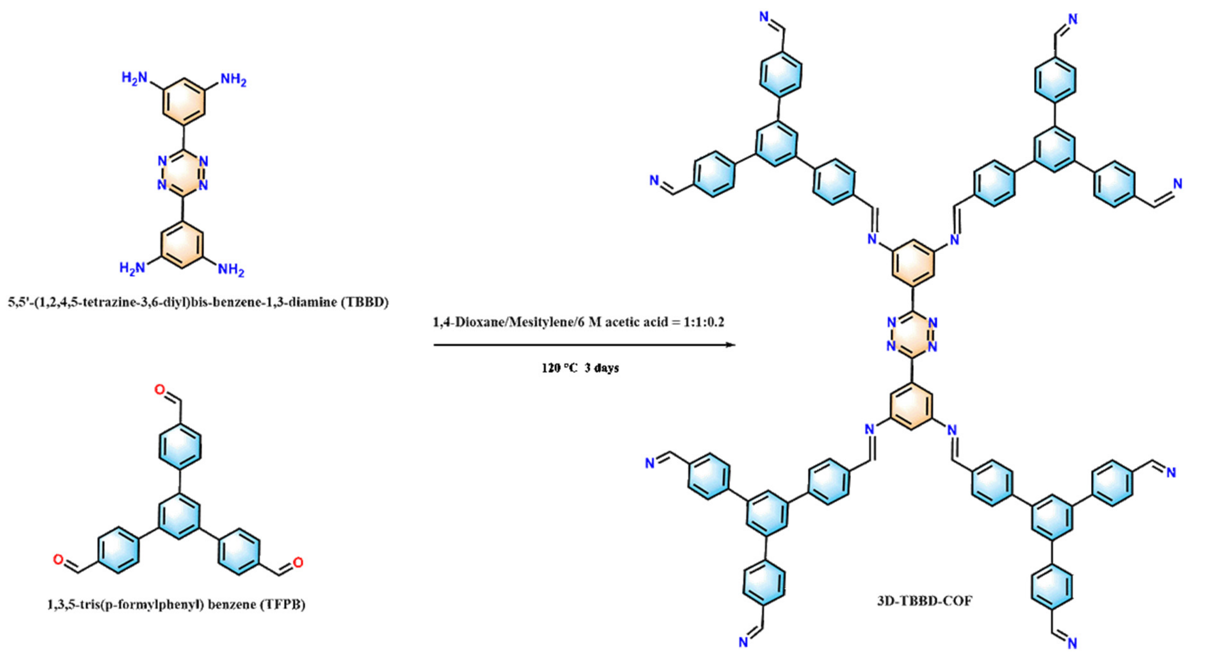

Supplementary Fig. 5 | Synthesis of 3D-TBBD-COF. The solvothermal reaction of TFPB with TBBD in 1,4-dioxnae, mesitylene, and 6 M acetic acid generates 3D-TBBD-COF.

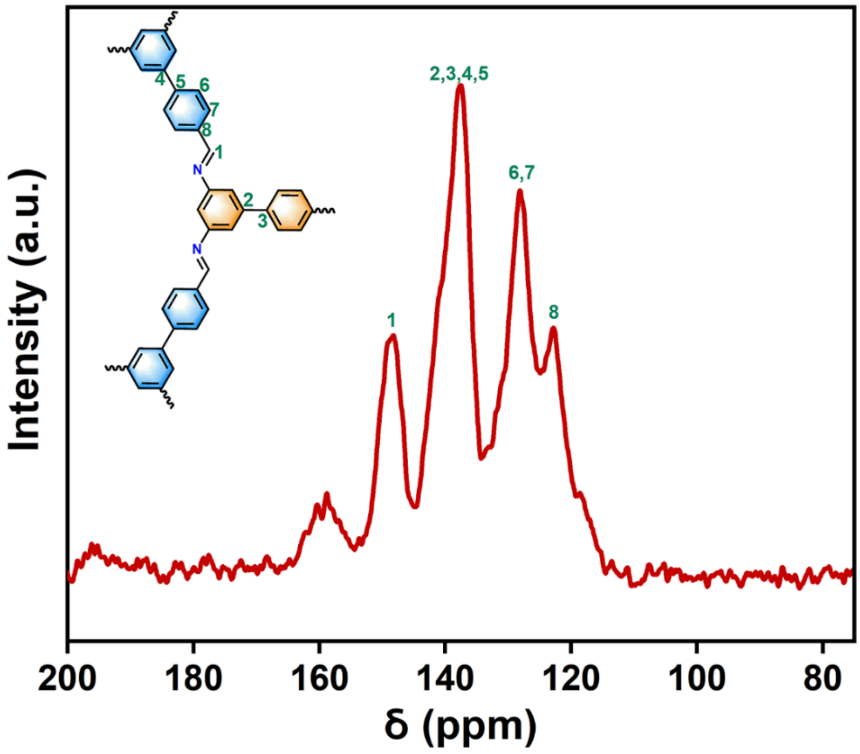

Supplementary Fig. 6 | ^{13}C ssNMR spectrum of 3D-TPT-COF. The characteristic C signal of the imine bond is observed at 148 ppm in the ^{13}C ssNMR spectrum of the 3D-TPT-COF, confirming the formation of imine bonds in 3D-TPT-COF.

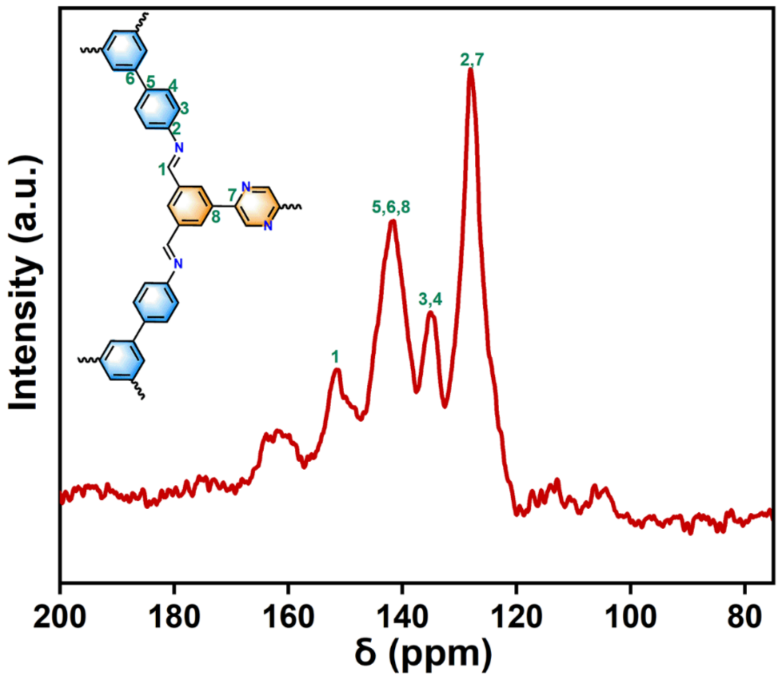

Supplementary Fig. 7 | ^{13}C ssNMR spectrum of 3D-PDDP-COF. The characteristic C signal of the imine bond is observed at 152 ppm in the ^{13}C ssNMR spectrum of the 3D-PDDP-COF, confirming the formation of imine bonds in 3D-PDDP-COF.

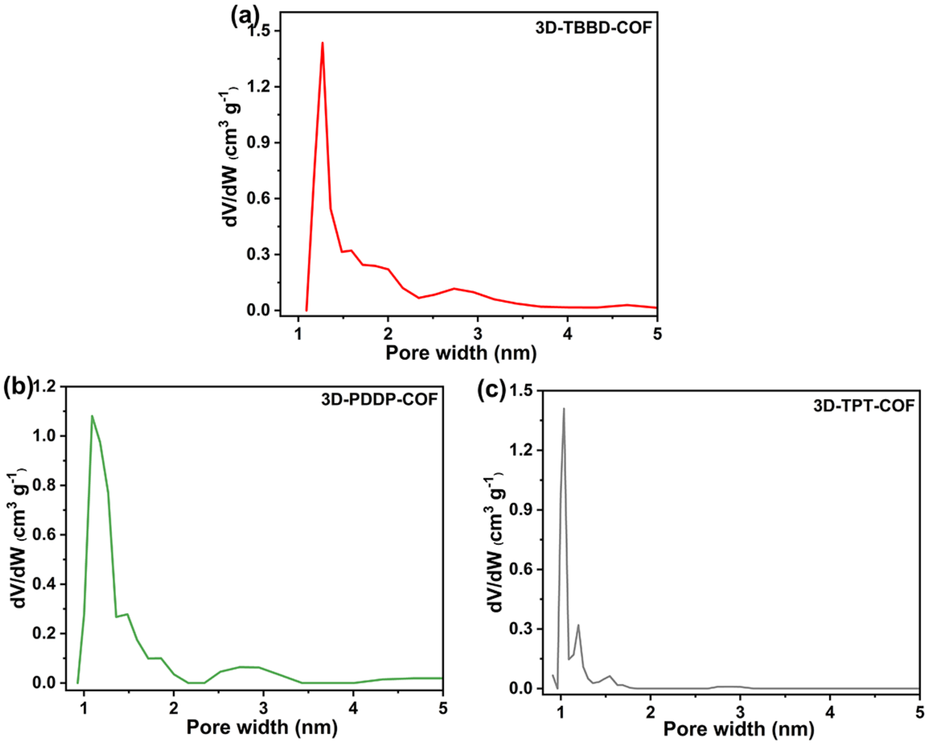

Supplementary Fig. 8 | Pore sizes of the three COFs. The pore sizes of **(a)** 3D-TBBD-COF, **(b)** 3D-PDDP-COF, and **(c)** 3D-TPT-COF calculated from nonlocal density functional theory are 10.5, 11.1, and 11.3 Å, respectively, revealing their microporous property.

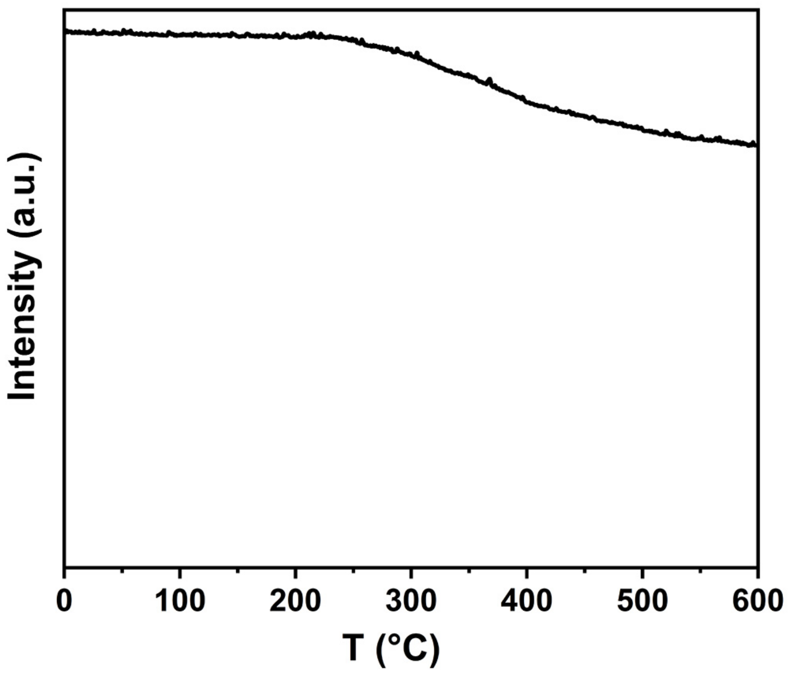

Supplementary Fig. 9 | TGA curve of 3D-TPT-COF. TGA discloses a decomposition temperature of *ca*. 320 °C for 3D-TPT-COF, proving its high thermal stability.

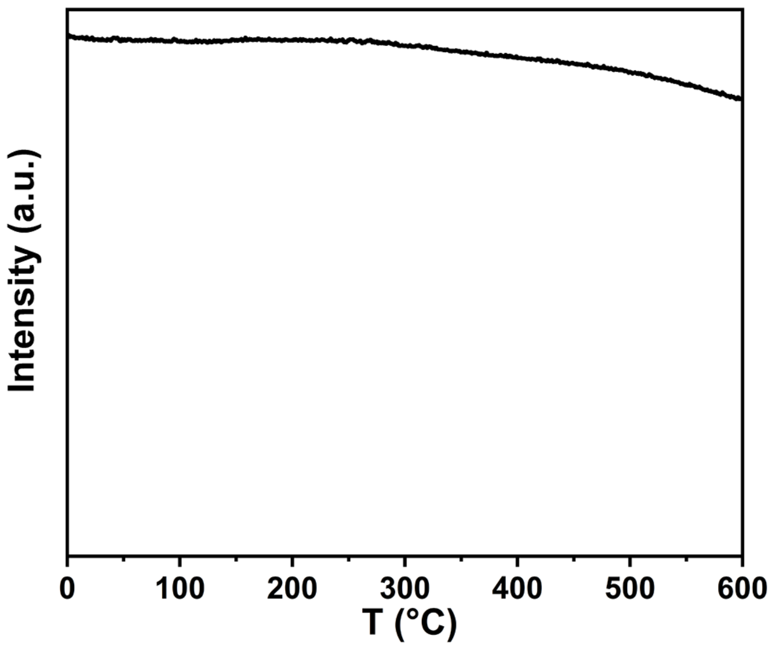

Supplementary Fig. 10 | TGA curve of 3D-PDDP-COF. TGA discloses a decomposition temperature of *ca*. 400 °C for the 3D-PDDP-COF, proving its high thermal stability.

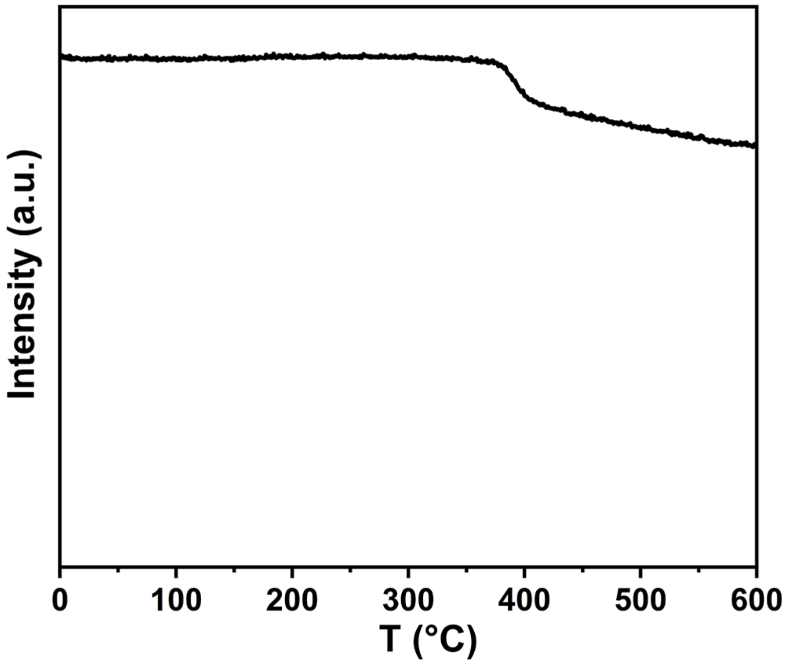

Supplementary Fig. 11 | TGA curve of 3D-TBBD-COF. TGA discloses a decomposition temperature of *ca*. 380 °C for the 3D-TBBD-COF, proving its high thermal stability

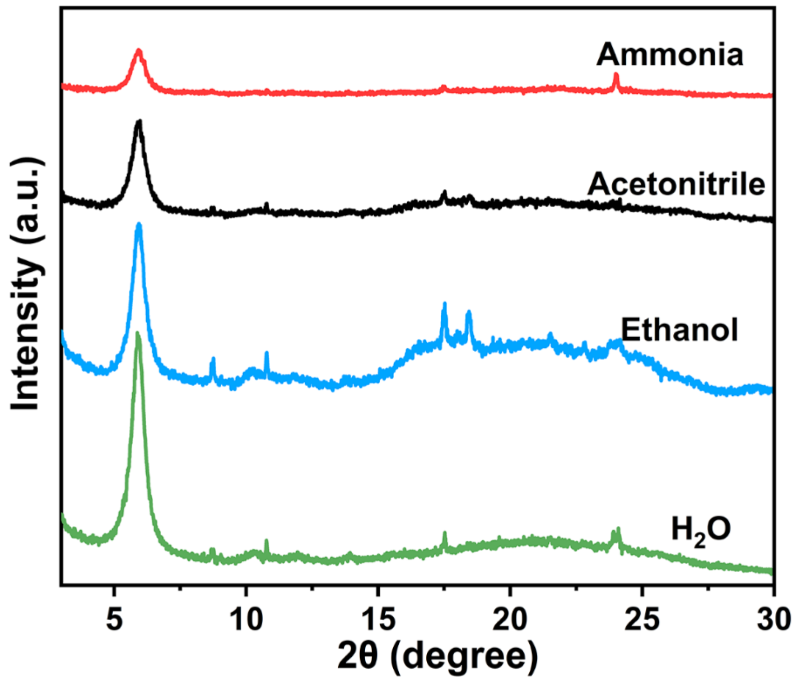

Supplementary Fig. 12 | PXRD patterns of 3D-TPT-COF. PXRD patterns of 3D-TPT-COF after treatment in different solvents: H_2O (green), acetonitrile (black), ethanol (blue), and ammonia (red) at 298 K.

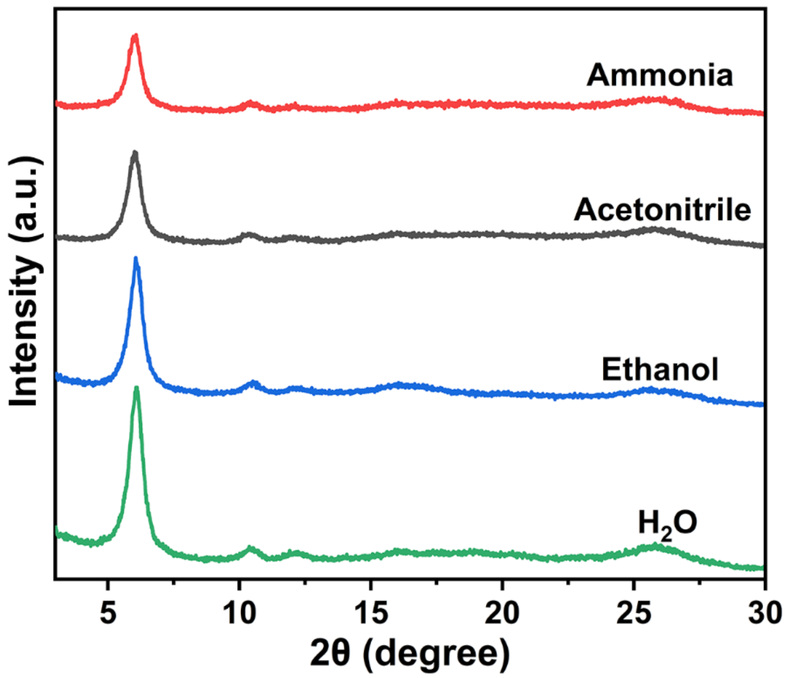

Supplementary Fig. 13 | PXRD patterns of 3D-PDDP-COF. PXRD patterns of 3D-PDDP-COF after treatment in different solvents: H_2O (green), acetonitrile (black), ethanol (blue), and ammonia (red) at 298 K.

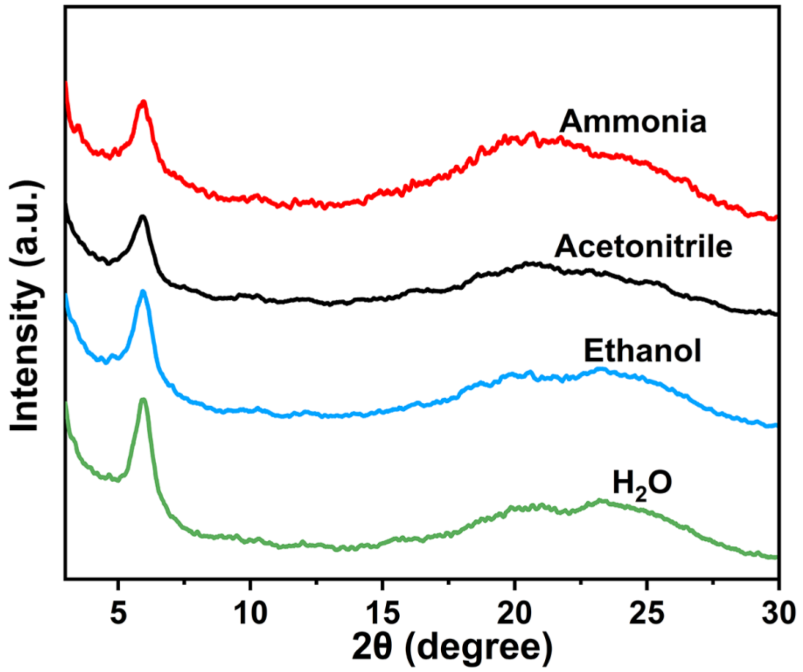

Supplementary Fig. 14 | PXRD patterns of 3D-TBBD-COF. PXRD patterns of 3D-TBBD-COF after treatment in different solvents: H_2O (green), acetonitrile (black), ethanol (blue), and ammonia (red) at 298 K.

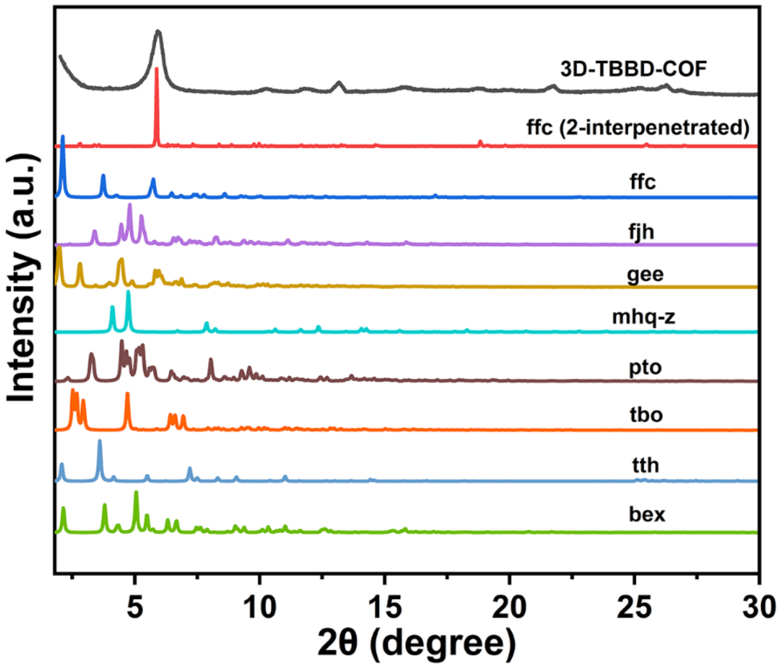

Supplementary Fig. 15 | Simulated PXRD patterns for other topologies for 3D-TBBD-COF. To determine their lattice packing, the PXRD of nine possible nets including pto (brown), tbo (orange), mhq-z (wathet), fjh (purple), gee (earthy yellow), ffc (blue), 2-ffc (red), bex (green), and tth (baby blue) topologies were constructed and optimized. As can be found, the experimentally revealed PXRD pattern of 3D-TBBD-COF matches well with the simulated one of the two-fold interpenetrated ffc topology.

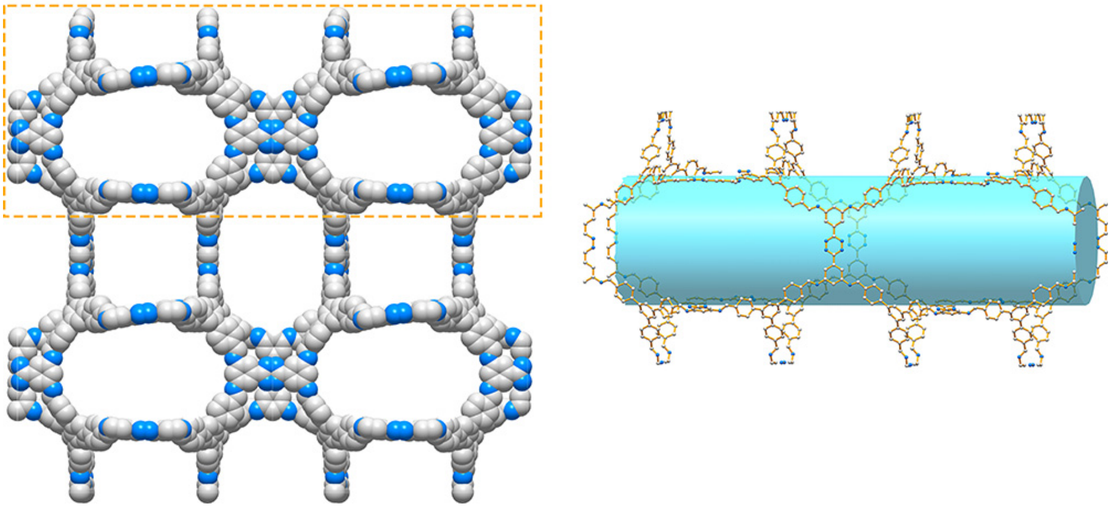

Supplementary Fig. 16 | Simulation structure of pto net for 3D-TBBD-COF. There are representatives of the 3D-TBBD-COF with pto net topologies. Grey and blue spheres represent C and N atoms, respectively. The blue translucent cylinders represent the pores of the 3D-TBBD-COF.

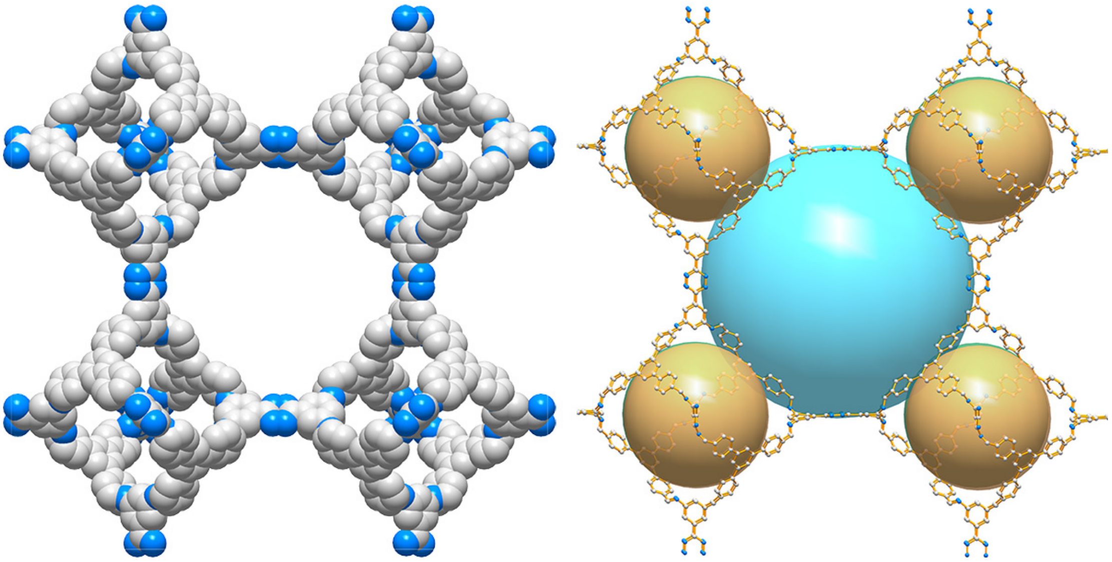

Supplementary Fig. 17 | Simulation structure of tbo net for 3D-TBBD-COF. There are representatives of the 3D-TBBD-COF with tbo net topologies. Grey and blue spheres represent C and N atoms, respectively. The blue and orange translucent spheres represent the pores of the 3D-TBBD-COF.

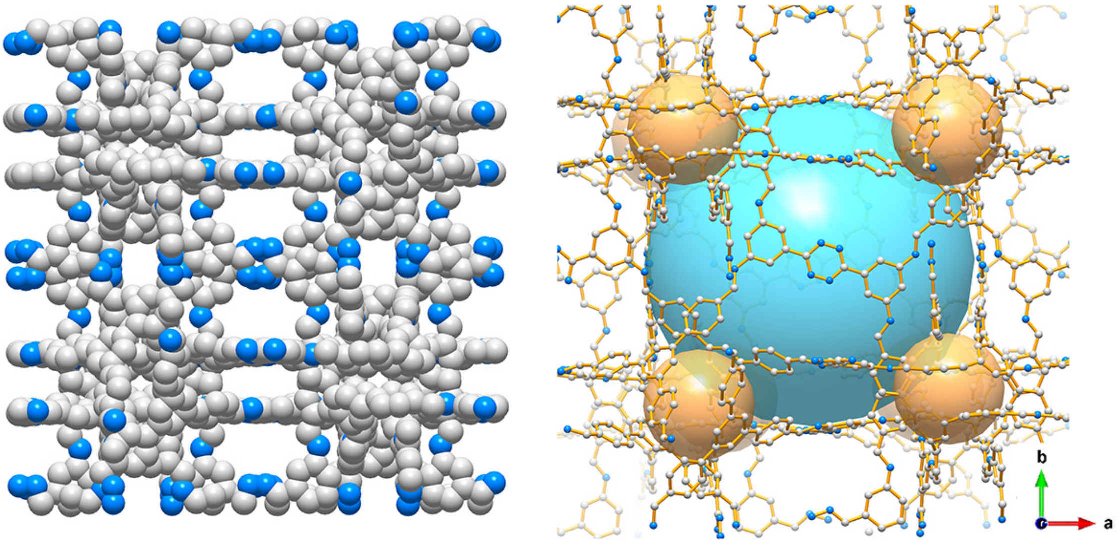

Supplementary Fig. 18 | Simulation structure of mhq-z net for 3D-TBBD-COF. There are representatives of the 3D-TBBD-COF with mhq-z net topologies. Grey and blue spheres represent C and N atoms, respectively. The blue and orange translucent spheres represent the pores of the 3D-TBBD-COF.

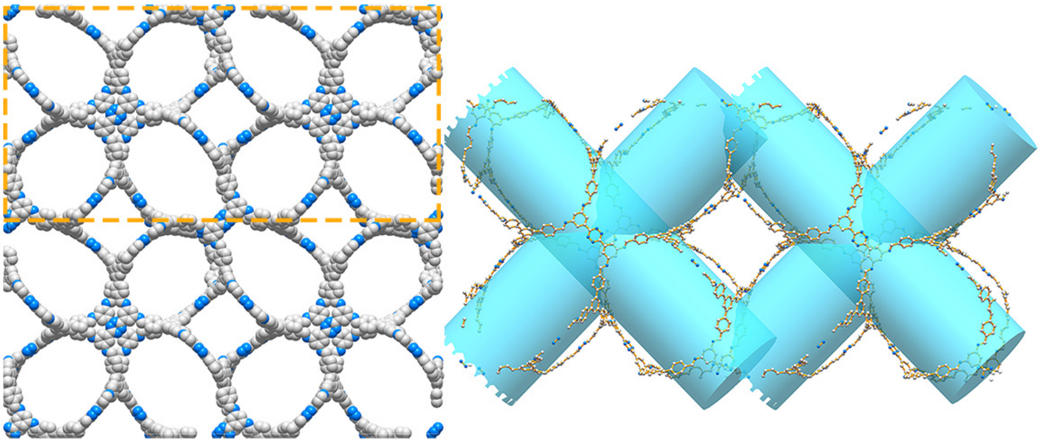

Supplementary Fig. 19 | Simulation structure of fjh net for 3D-TBBD-COF. There are representatives of the 3D-TBBD-COF with fjh net topologies. Grey and blue spheres represent C and N atoms, respectively. The blue translucent cylinders represent the pores of the 3D-TBBD-COF.

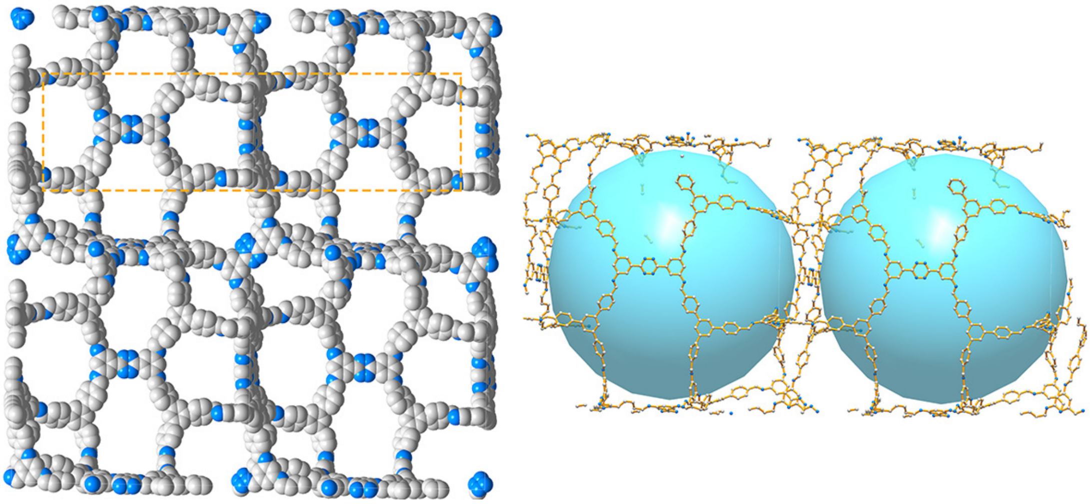

Supplementary Fig. 20 | Simulation structure of gee net for 3D-TBBD-COF. There are representatives of the 3D-TBBD-COF with gee net topologies. Grey and blue spheres represent C and N atoms, respectively. The blue translucent cylinders represent the pores of the 3D-TBBD-COF.

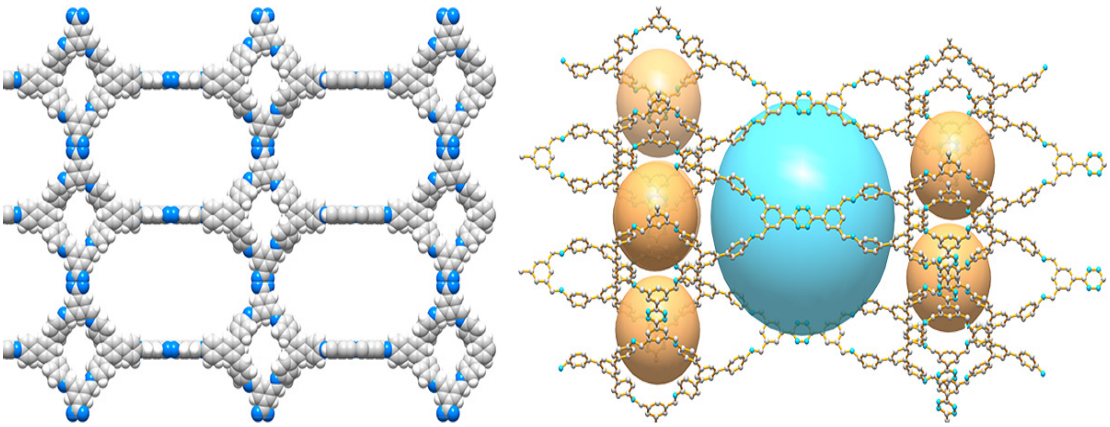

Supplementary Fig. 21 | Simulation structure of ffc net for 3D-TBBD-COF. There are representatives of the 3D-TBBD-COF with ffc net topologies. Grey and blue spheres represent C and N atoms, respectively. The blue and orange translucent cylinders represent the pores of the 3D-TBBD-COF.

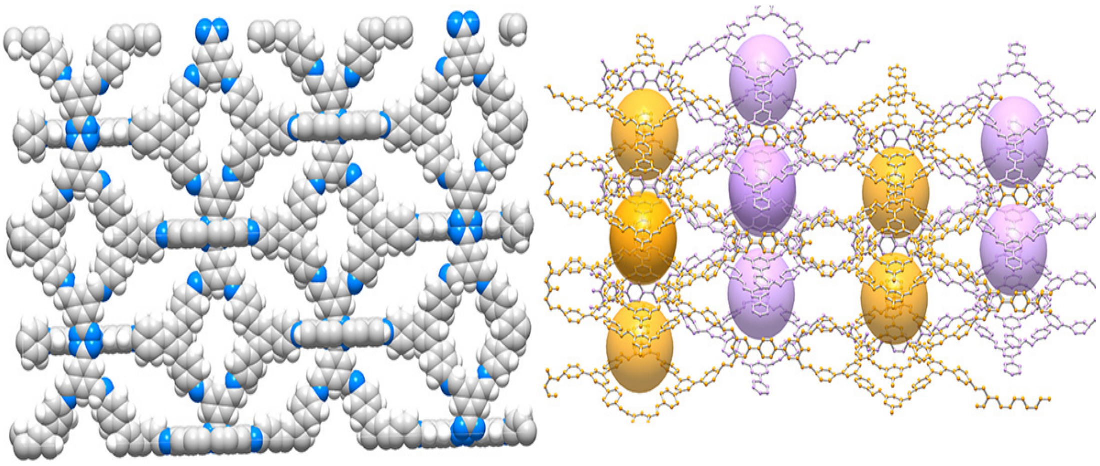

Supplementary Fig. 22 | Simulation structure of two-fold interpenetrated ffc net for 3D-TBBD-COF. There are representatives of the 3D-TBBD-COF with two-fold interpenetrated ffc net topologies. Grey and blue spheres represent C and N atoms, respectively. The purple and orange translucent cylinders represent the pores of the 3D-TBBD-COF.

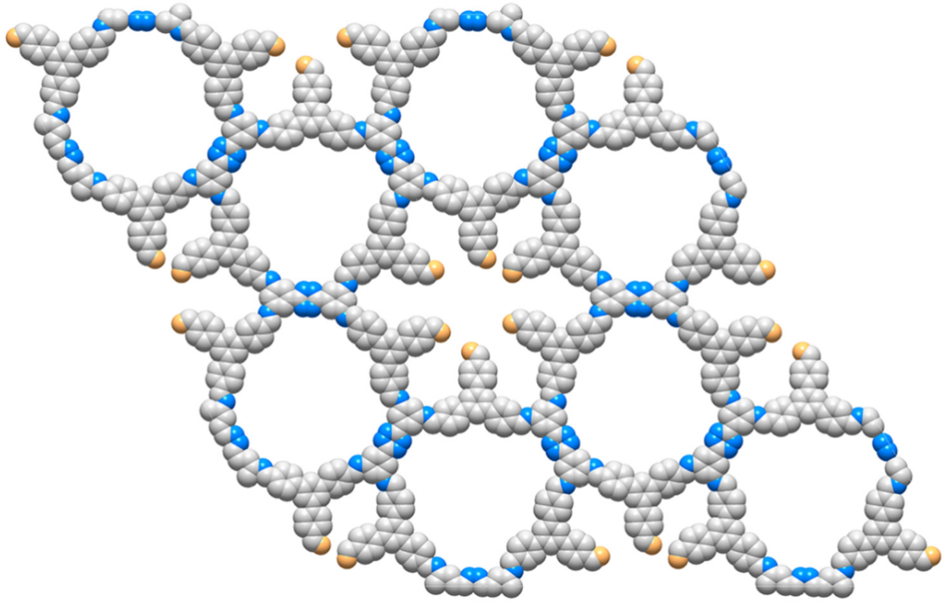

Supplementary Fig. 23 | Simulation structure of substoichiometric 2D tth net for 3D-TBBD-COF. There are representatives of the 3D-TBBD-COF with 2D tth net topologies. Grey, orange, and blue spheres represent C, O, and N atoms, respectively.

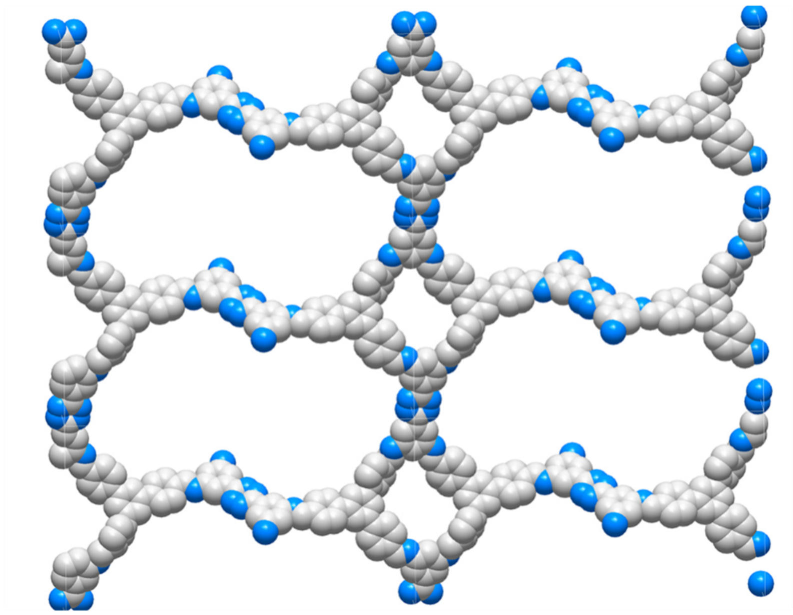

Supplementary Fig. 24 | Simulation structure of substoichiometric 2D bex net for 3D-TBBD-COF. There are representatives of the 3D-TBBD-COF with 2D bex net topologies. Grey and blue spheres represent C and N atoms, respectively.

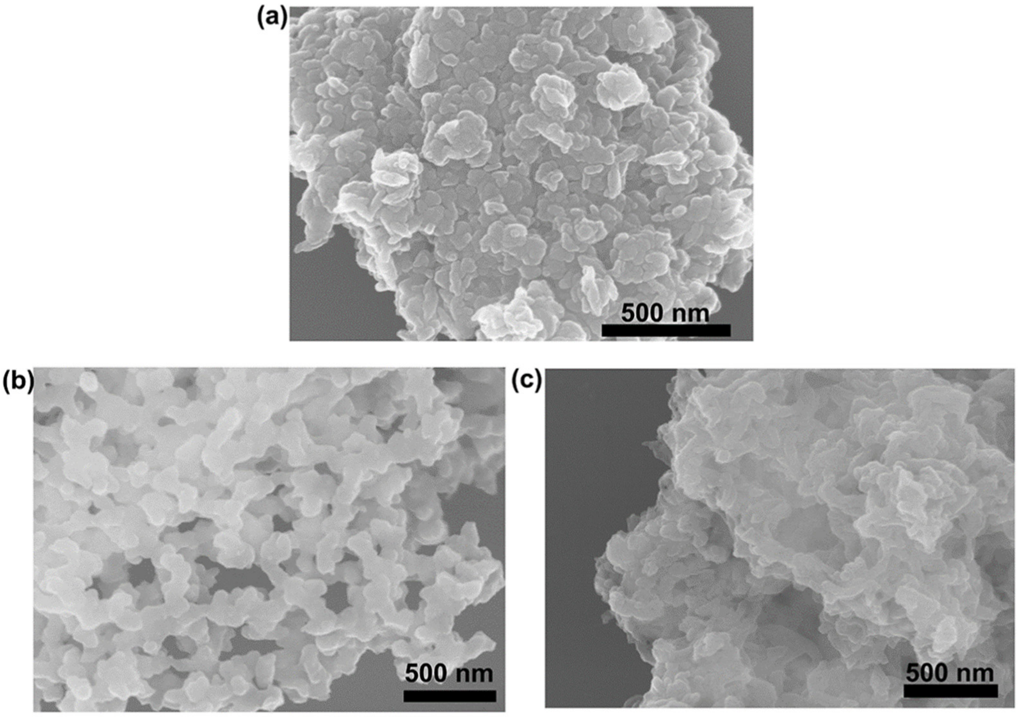

Supplementary Fig. 25 | SEM images of 3D-TPT-COF, 3D-PDDP-COF, and 3D-TBBD-COF. SEM was used to analyze the morphology of **(a)** 3D-TPT-COF, **(b)** 3D-PDDP-COF, and **(c)** 3D-TBBD-COF. SEM images disclose their irregular spherical shapes with a size of *ca*. 300-500 nm.

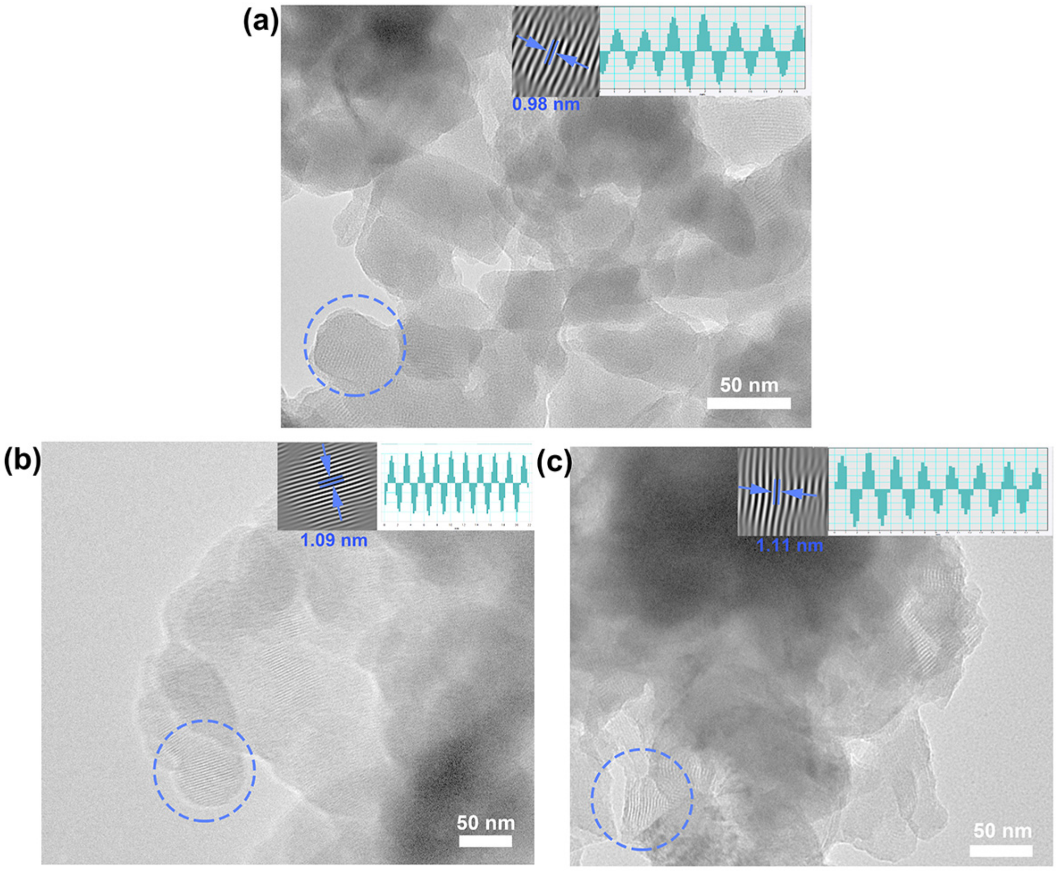

Supplementary Fig. 26 | HR-TEM images of 3D-TPT-COF, 3D-PDDP-COF, and 3D-TBBD-COF. HR-TEM images exhibit distinct lattice stripes of **(a)** 3D-TPT-COF, **(b)** 3D-PDDP-COF, and **(c)** 3D-TBBD-COF with a spacing of 0.98, 1.09, and 1.11 nm, respectively, corresponding to their (101) bragg peaks, proving their good crystalline nature.

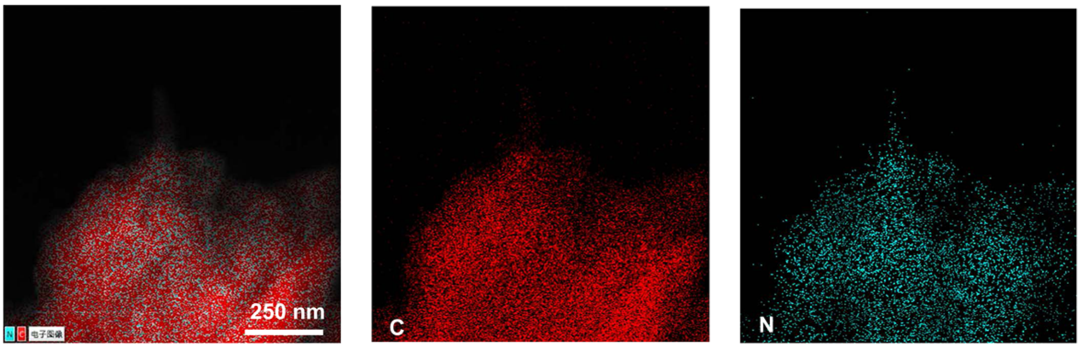

Supplementary Fig. 27 | EDS mapping images of 3D-TBBD-COF. EDS mapping images reveal the uniformly distributed C and N elements over the 3D-TBBD-COF sample.

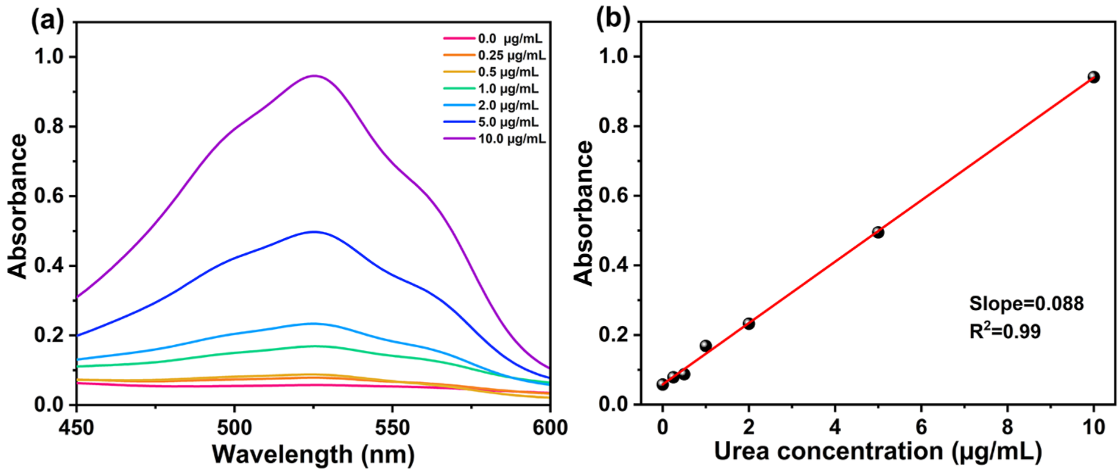

Supplementary Fig. 28 | Detection of urea. (a) UV-vis spectra and **(b)** calibration curve for quantification of urea in diacetylmonoxime method. As-produced urea was spectrophotometrically determined by diacetylmonoxime method. The concentration–absorbance curves were calibrated using standard urea solutions, which contained the same concentrations of electrolytes as used in the electrocatalysis experiments.

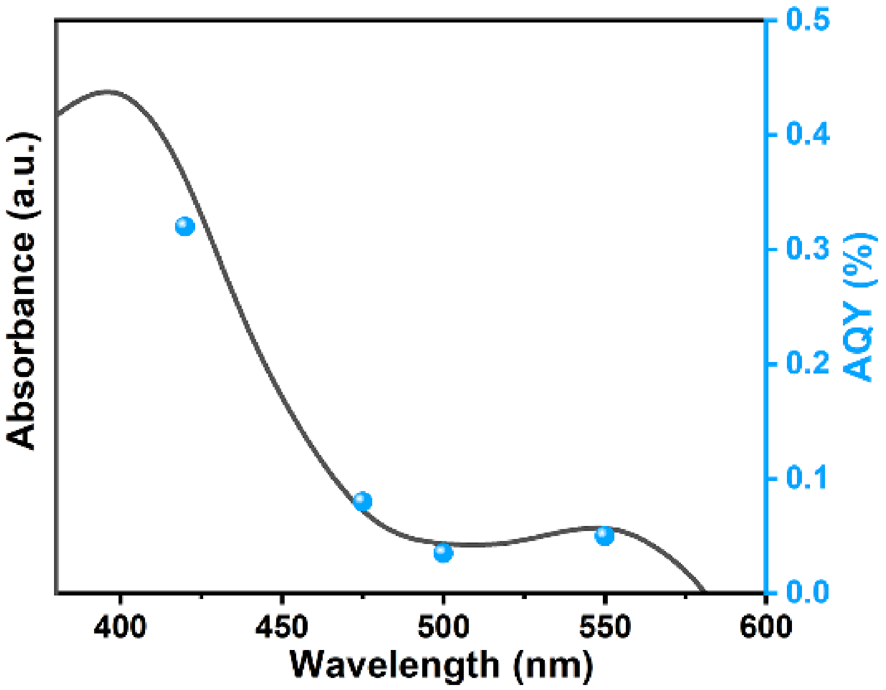

Supplementary Fig. 29 | The AQY (Blue dot) and UV/vis absorption spectra (black curve) of 3D-TBBD-COF. The AQY of the photocatalyst was measured under 300 W Xe lamp irradiation (equipped with bandpass filters of 420, 475, 500, and 550 nm). The AQY for 3D-TBBD-COF was 0.32 % observed at 420 nm.

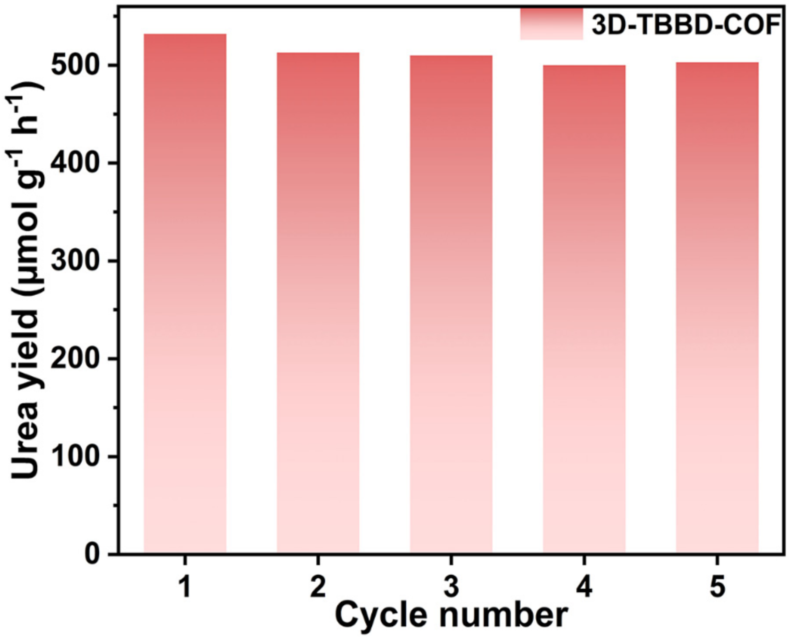

Supplementary Fig. 30 | Cyclic stability of 3D-TBBD-COF for photocatalytic urea synthesis. 3D-TBBD-COF was separated from the mixture at the end of the reaction by centrifugation. The solid was washed and subsequently reused for the next cycle. The same procedure was repeated for five cycles to assess the recyclability of this catalyst. The urea yield of 3D-TBBD-COF remains almost constant after five consecutive cycles of the reaction, revealing the excellent photostability of 3D-TBBD-COF.

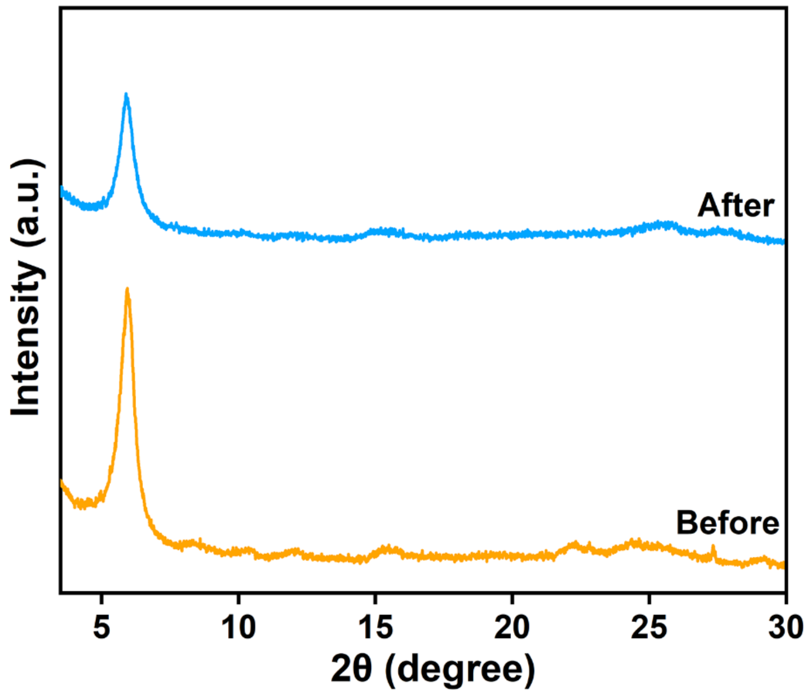

Supplementary Fig. 31 | PXRD patterns of 3D-TBBD-COF before and after photocatalytic reactions. The recycled 3D-TBBD-COF sample after photocatalytic cycles exhibits a very similar PXRD pattern to those for the 3D-TBBD-COF sample before photocatalytic cycles, confirming its excellent stability during the photocatalytic reaction process.

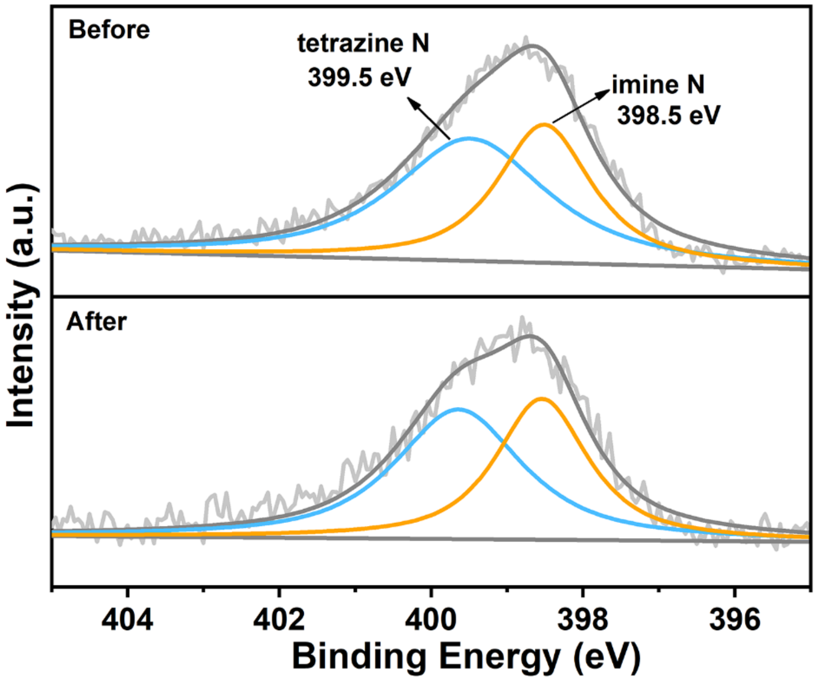

Supplementary Fig. 32 | XPS N *1s* spectra of 3D-TBBD-COF before and after photocatalytic reactions. The recycled 3D-TBBD-COF sample after photocatalytic cycles exhibits a very similar XPS N *1s* spectra to those for the 3D-TBBD-COF sample before photocatalytic cycles, confirming its excellent stability during the photocatalytic reaction process.

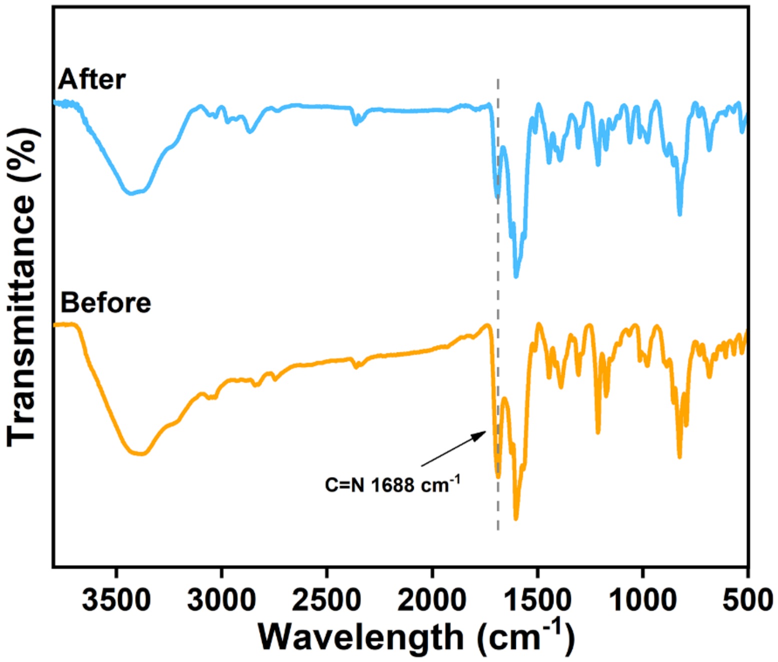

Supplementary Fig. 33 | FT-IR spectra of 3D-TBBD-COF before and after photocatalytic reaction. The recycled 3D-TBBD-COF sample after photocatalytic cycles exhibits a very similar FT-IR spectrum to those for the 3D-TBBD-COF sample before photocatalytic cycles, confirming its excellent stability during the photocatalytic reaction process.

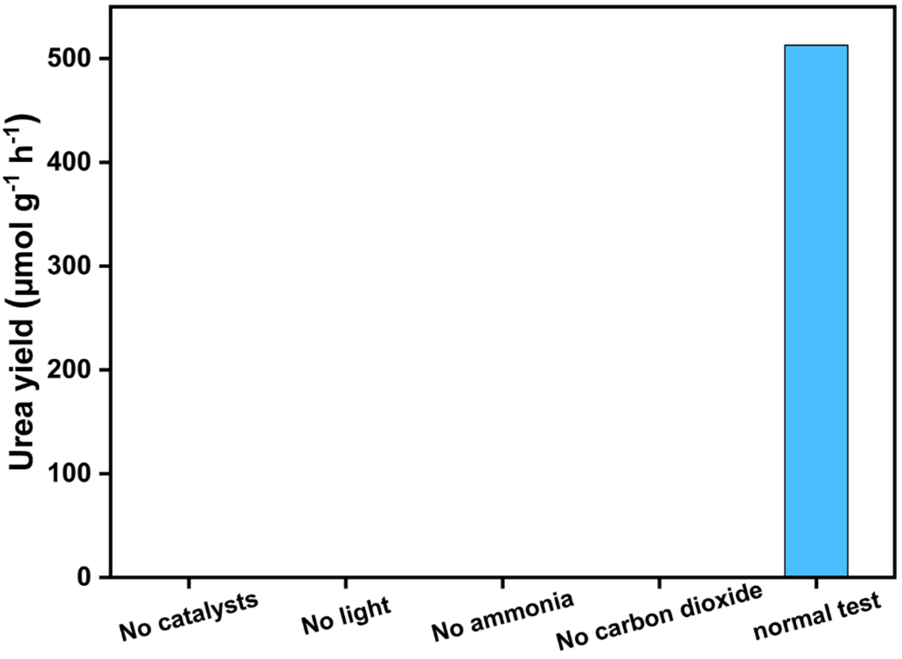

Supplementary Fig. 34 | The urea yields under different experimental conditions. To identify the role of 3D-TBBD-COF in the photocatalytic system, various control experiments were carried out. In the absence of COF photocatalysts, no urea was generated in the reactor, manifesting the photocatalyst nature of 3D-TBBD-COF in photocatalytic NH_3 and CO_2 coupling reaction to produce urea. As expected, the urea production reaction carried out in the dark gives no target product, disclosing the light irradiation promotion nature for the urea production from NH_3 and CO_2. Nevertheless, no urea was detected without adding NH_3 or CO_2 into the reactor, revealing the origination of the urea from the coupling reaction between NH_3 and CO_2.

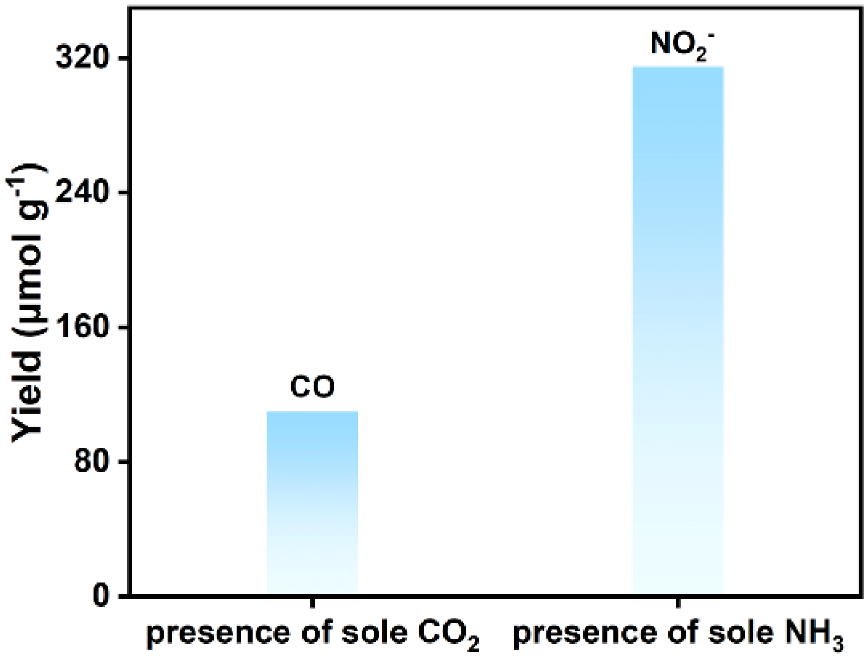

Supplementary Fig. 35 | The photocatalytic products of 3D-TBBD-COF in the presence of sole CO_2 and sole NH_3. Photocatalytic tests for the 3D-TBBD-COF in the presence of sole CO_2 or NH_3 have been performed. CO with a yield of 110 μmol g^{-1} was determined in the presence of sole CO_2 while NO_2^- with a yield of 315 μmol g^{-1} was found in the presence of sole NH_3, demonstrating the photocatalytic activity of TBBD towards CO_2 reduction and NH_3 oxidation.

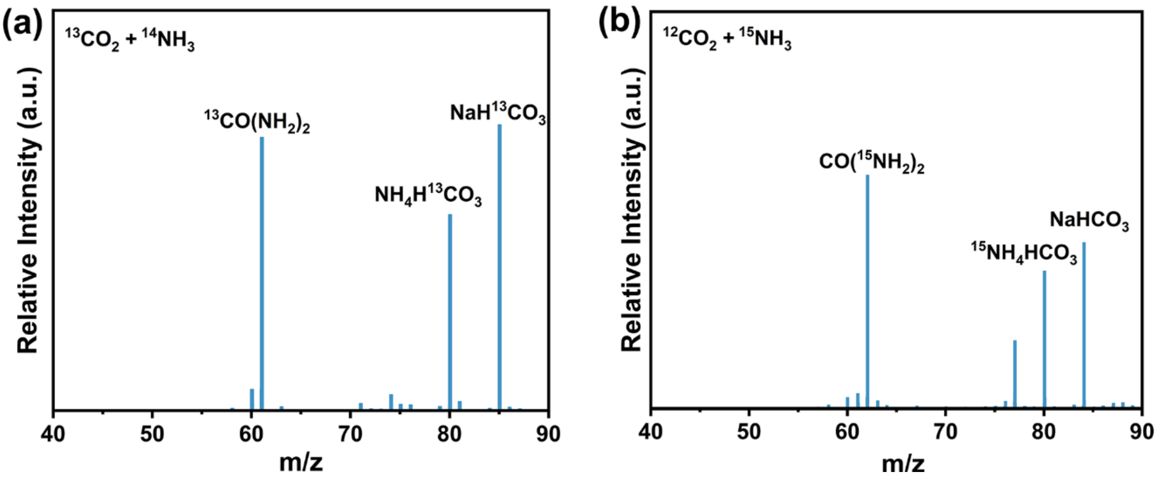

Supplementary Fig. 36 | HR-MS spectra of 3D-TBBD-COF photosynthetic urea. HR-MS spectra of 3D-TBBD-COF photocatalysis **(a)** $^{13}CO_2$+$^{14}NH_3$ and **(b)** $^{12}CO_2$+$^{15}NH_3$ synthesis systems. To confirm the nitrogen and carbon sources of urea generated, photocatalytic experiments were performed with $^{15}NH_3$ and $^{13}CO_2$ as the feedstock, respectively. As can be seen, the employment of $^{15}NH_3$ and $^{13}CO_2$ as the reactant leads to the observation of a signal at m/z = 62 and 61, respectively, in the HR-MS spectra, corresponding to the ^{15}N and ^{13}C labeled urea molecules, confirming the urea origination from NH_3 and CO_2 coupling reaction. This is further confirmed by the observation of the signals at m/z = 80 and 85 due to ^{13}C-labeled $NH_4H^{13}CO_3$ and ^{13}C-labeled $NaH^{13}CO_3$ for $^{13}CO_2$+$^{14}NH_3$ system and m/z = 80 and 84 due to ^{15}N-labeled $^{15}NH_4HCO_3$ and $NaHCO_3$ for the $^{12}CO_2$+$^{15}NH_3$ system.

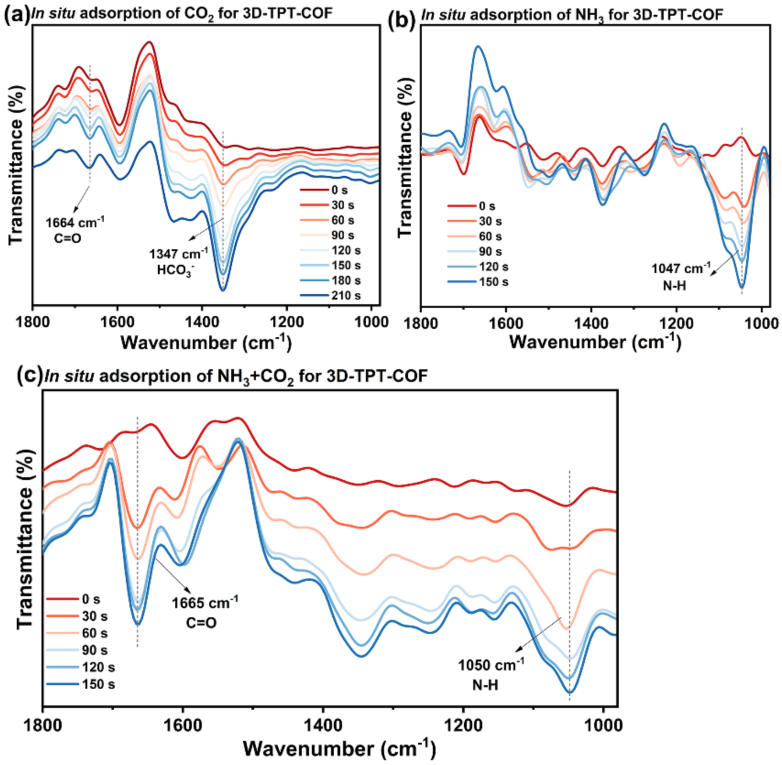

Supplementary Fig. 37 | *In situ* adsorption DRIFT spectra for 3D-TPT-COF. *In situ* DRIFT spectra for adsorption of **(a)** CO_2, **(b)** NH_3, and **(c)** CO_2+NH_3 in water on 3D-TPT-COF. The peaks at 1664 and 1347 cm^{-1} due to the C=O and HCO_3^- stretching bands of the adsorbed CO_2 molecules get appeared and increased along with the increase of adsorption time in the *in-situ* DRIFT spectra for adsorption of CO_2 and CO_2+NH_3 in water on 3D-TPT-COF, while new band appeared at 1047 cm^{-1} is due to the N-H stretching and bending vibration of the adsorbed NH_3 in the *in-situ* DRIFT spectra for adsorption of NH_3 and CO_2+NH_3 in water on 3D-TPT-COF.

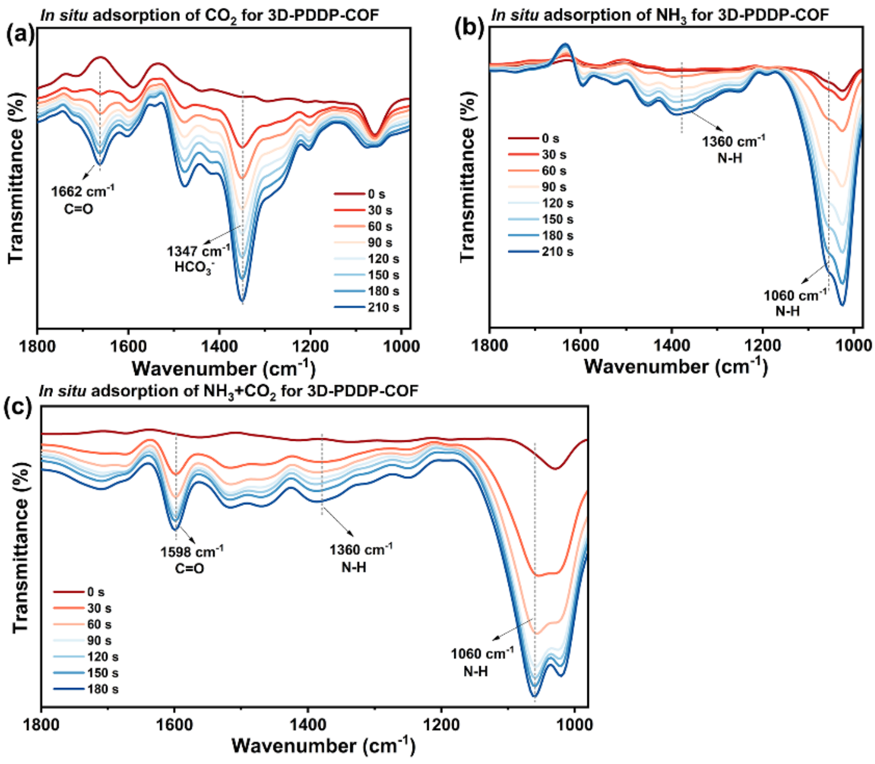

Supplementary Fig. 38 | *In situ* adsorption DRIFT spectra of 3D-PDDP-COF. *In situ* DRIFT spectra for adsorption of **(a)** CO_2, **(b)** NH_3, and **(c)** CO_2+NH_3 in water on 3D-PDDP-COF. The peaks at 1662 and 1347 cm^{-1} due to the C=O and HCO_3^- stretching bands of the adsorbed CO_2 molecules get appeared and increased along with the increase of adsorption time in the *in-situ* DRIFT spectra for adsorption of CO_2 and CO_2+NH_3 in water on 3D-PDDP-COF, while new band appeared at 1060 cm^{-1} is due to the N-H stretching and bending vibration of the adsorbed NH_3 in the *in-situ* DRIFT spectra for adsorption of NH_3 and CO_2+NH_3 in water on3D-PDDP-COF.

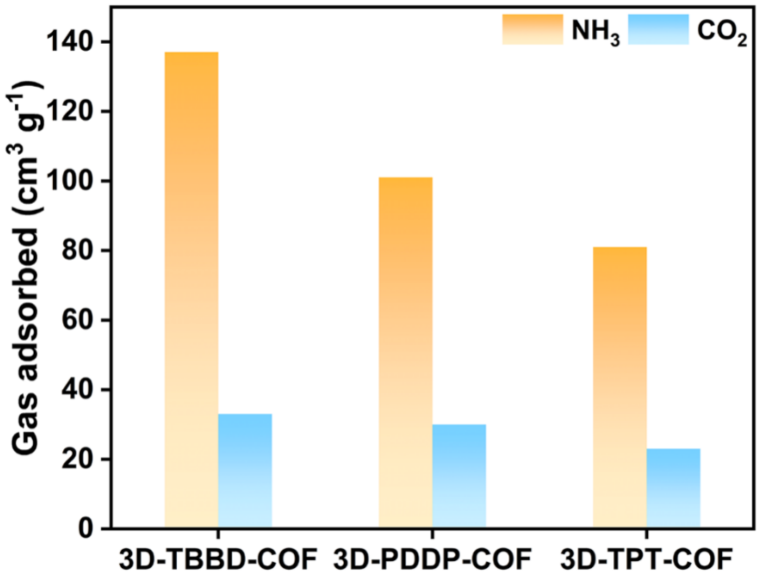

Supplementary Fig. 39 | CO_2 and NH_3 adsorption capacity of three COFs. CO_2 and NH_3 adsorption capacity of 3D-TPT-COF, 3D-PDDP-COF, and 3D-TBBD-COF using the static volumetric method at 298 K. Gas adsorption experiment results reveal the higher adsorption capacity of 3D-TBBD-COF to both NH_3 (131 cm^3 g^{-1}) and CO_2 (33 cm^3 g^{-1}) at room temperature compared to 3D-TPT-COF (86 and 24 cm^3 g^{-1}) and 3D-PDDP-COF (101 and 30 cm^3 g^{-1}) owing to the more exposed lone pair electrons in the tetrazine-containing framework of 3D-TBBD-COF over the benzene-containing 3D-TPT-COF and pyridine-containing 3D-PDDP-COF.

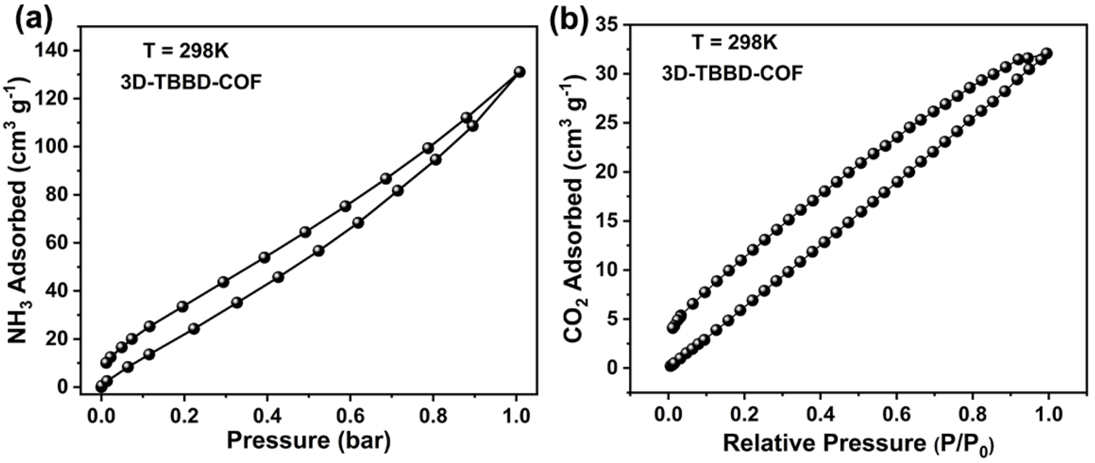

Supplementary Fig. 40 | CO_2 and NH_3 adsorption capacity of 3D-TBBD-COF. (a) NH_3 and **(b)** CO_2 adsorption-desorption isotherms for 3D-TBBD-COF at 298 K. The NH_3 and CO_2 uptakes of 3D-TBBD-COF at $P/P_0 = 1.0$ under room temperature amount to 131 and 33 $cm^3\ g^{-1}$, respectively.

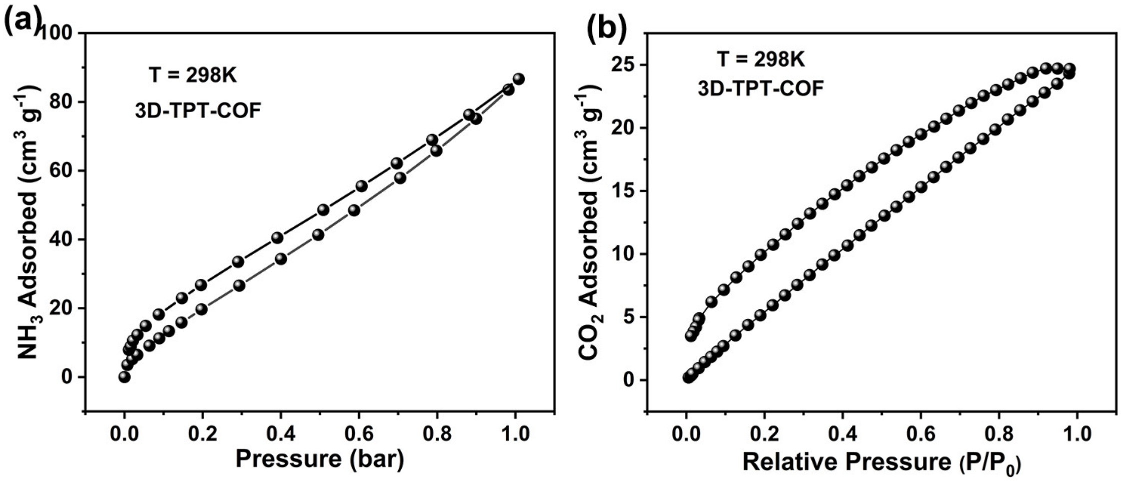

Supplementary Fig. 41 | CO_2 and NH_3 adsorption capacity of 3D-TPT-COF. (a) NH_3 and **(b)** CO_2 adsorption-desorption isotherms for 3D-TPT-COF at 298 K. The NH_3 and CO_2 uptakes of 3D-TPT-COF at $P/P_0 = 1.0$ under room temperature amount to 86 and 24 $cm^3\ g^{-1}$, respectively.

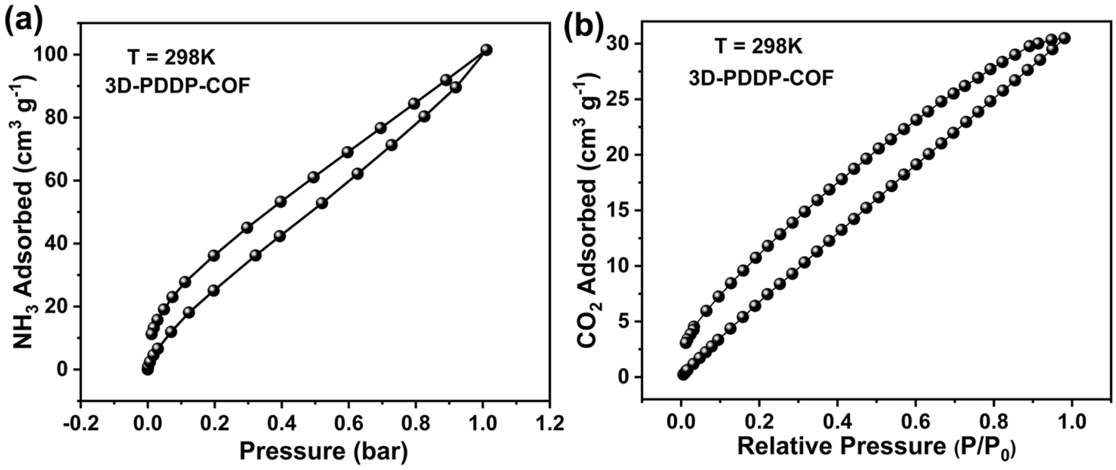

Supplementary Fig. 42 | CO_2 and NH_3 adsorption capacity of 3D-PDDP-COF. (a) NH_3 and **(b)** CO_2 adsorption-desorption isotherms for 3D-PDDP-COF at 298 K. The NH_3 and CO_2 uptakes of 3D-PDDP-COF at $P/P_0 = 1.0$ under room temperature amount to 101 and 30 cm^3 g^{-1}, respectively.

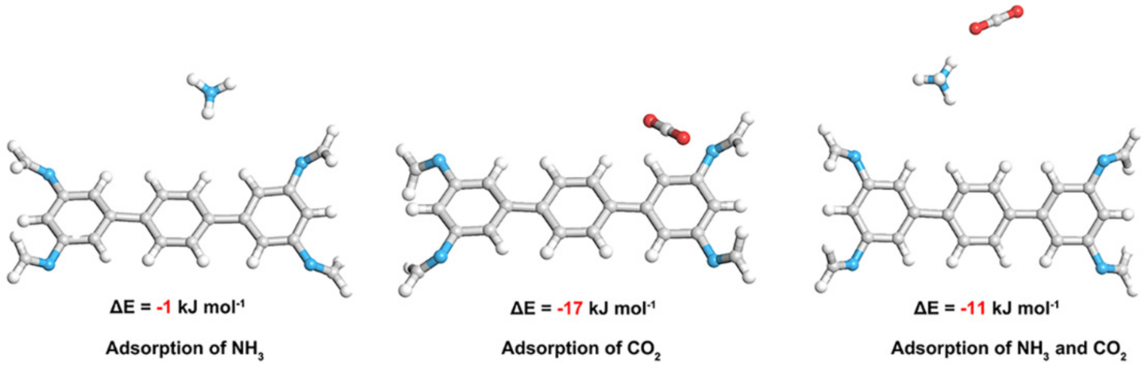

Supplementary Fig. 43 | Adsorption structures and energies for NH_3, CO_2, and CO_2+NH_3 on 3D-TPT-COF. On the basis of the adsorption energy calculations at the level of M06-2X/6-311G(d), the adsorption energy for NH_3, CO_2, and CO_2+NH_3 on 3D-TPT-COF amounts to -1, -17, and -11 kJ mol^{-1}, respectively. Grey, red, white, and blue spheres represent C, O, H, and N atoms, respectively.

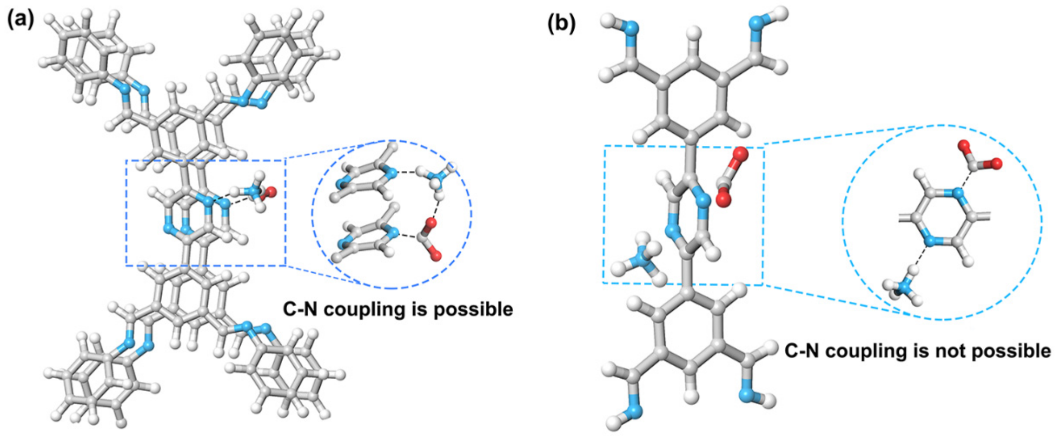

Supplementary Fig. 44 | Schematic diagram of 3D-PDDP-COF photocatalytic coupling. Schematic representation of the **(a)** possible and **(b)** impossible implementation of 3D-PDDP-COF for the adsorption and photocatalytic generation of urea. For 3D-PDDP-COF, the two reactants, CO_2 and NH_4^+, can not depend on the two oppositely arranged N atoms in one pyrazine moiety with N····N distance of ~2.6 Å to proceed with the C-N coupling, Supplementary Fig. 44b. Fortunately, these two reactants are able to depend on two pyrazine N atoms in neighboring layers in the framework with N····N distance of ~3.5 Å to complete the C-N coupling, Supplementary Fig. 44a. This, however, requires an obvious energy barrier of 2.00 eV, leading to a significantly weakened photocatalytic CO_2 and NH_3 coupling efficiency. Grey, red, white, and blue spheres represent C, O, H, and N atoms, respectively.

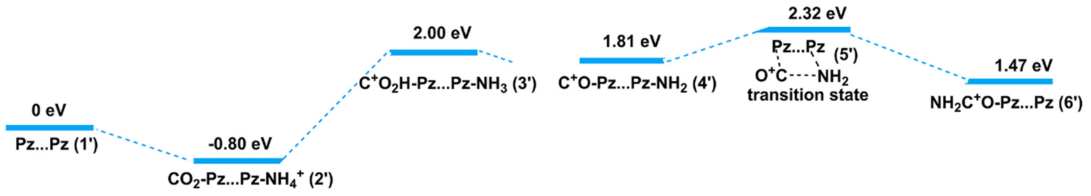

Supplementary Fig. 45 | The free energy for generation of half urea form CO_2 and NH_3 on 3D-PDDP-COF. To clarify the photocatalytic mechanism for the urea generation from CO_2 and NH_3 on the COFs, the Gibbs free energy calculations were carried out at the level of M06-2X/6-311G(d). The photosensitizer Pz captures a photon with a wavelength in the range of 200-600 nm, the reaction will start from the hydrogen transfer from NH_4^+ to CO_2 along the heptatomic ring. Subsequently, the key intermediates of C^+O_2H-Pz...Pz-NH_3 were further generated with an energy barrier of 2.00 eV. The next step involves the dehydration process to produce two unstable intermediate species *C^+O and *NH_2 with an unpaired electron. In the next moment, *C^+O and *NH_2 will combine into the half urea NH_2C^+O, completing the C-N coupling process.

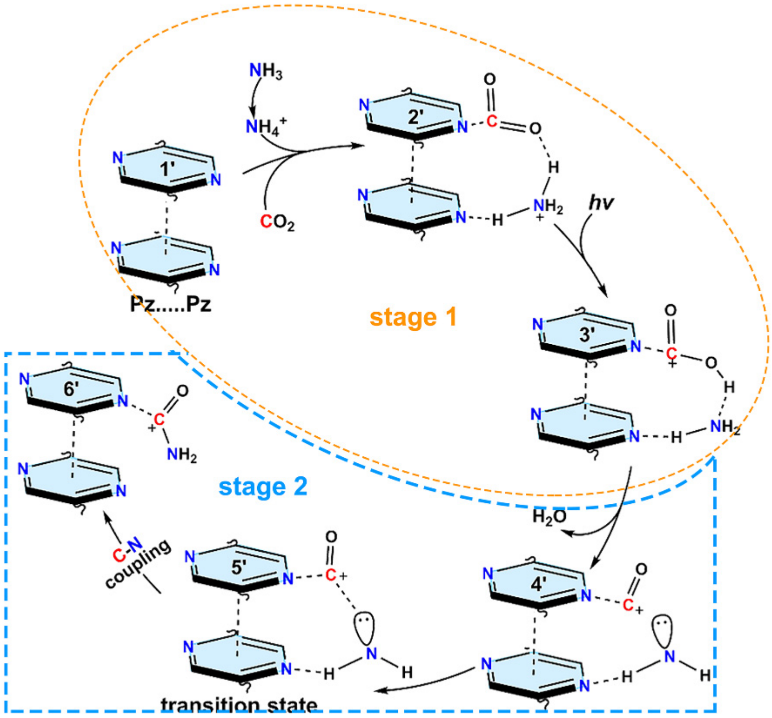

Supplementary Fig. 46 | The reaction pathway for generation of half urea form CO_2 and NH_3 on 3D-PDDP-COF. Based on the calculation results, the catalytic route on 3D-PDDP-COF could be divided into two stages: **(1)** Photon capture and generation of excited pyrazine moiety (Pz*); **(2)** The first C-N coupling process is realized by means of the electron absorption induction effect of multiple N atoms of Pz and Pz molecules.

References

S1. Dey, A.; Pradhan, J.; Biswas, S.; Ahamed Rahimi, F.; Biswas, K.; Maji, T. K., COF-Topological Quantum Material Nano-heterostructure for CO_2 to Syngas Production under Visible Light. *Angew. Chem., Int. Ed.* **63**, e202315596 (2024).

S2. Liu, W.; Wu, E.; Yu, B.; Liu, Z.; Wang, K.; Qi, D.; Li, B.; Jiang, J., Reticular Synthesis of Metal-Organic Frameworks by 8-Connected Quadrangular Prism Ligands for Water Harvesting. *Angew. Chem., Int. Ed.* **62**, e202305144 (2023).

S3. Hu, Q.; Zhou, W.; Qi, S.; Huo, Q.; Li, X.; Lv, M.; Chen, X.; Feng, C.; Yu, J.; Chai, X.; Yang, H.; He, C., Pulsed co-electrolysis of carbon dioxide and nitrate for sustainable urea synthesis. *Nat. Sustain.* **7**, 442-451 (2024).

S4. Huang, Y.; Yang, R.; Wang, C.; Meng, N.; Shi, Y.; Yu, Y.; Zhang, B., Direct Electrosynthesis of Urea from Carbon Dioxide and Nitric Oxide. *ACS Energy Lett.* **7**, 284-291 (2022).

S5. Luo, Y.; Xie, K.; Ou, P.; Lavallais, C.; Peng, T.; Chen, Z.; Zhang, Z.; Wang, N.; Li, X.-Y.; Grigioni, I.; Liu, B.; Sinton, D.; Dunn, J. B.; Sargent, E. H., Selective electrochemical synthesis of urea from nitrate and CO_2 via relay catalysis on hybrid catalysts. *Nat. Catal.* **6**, 939-948 (2023).

S6. Zhao, Y.; Truhlar, D. G., The M06 suite of density functionals for main group thermochemistry, thermochemical kinetics, noncovalent interactions, excited states, and transition elements: two new functionals and systematic testing of four M06-class functionals and 12 other functionals. *Theor. Chem. Acc.* **120**, 215-241 (2008).

S7. M. J. Frisch, G. W. Trucks, H. B. Schlegel, G. E. Scuseria, M. A. Robb, J. R. Cheeseman, G. Scalmani, V. Barone, G. A. Petersson, H. Nakatsuji, X. Li, M. Caricato, A. V. Marenich, J. Bloino, B. G. Janesko, R. Gomperts, B. Mennucci, H. P. Hratchian, J. V. Ortiz, A. F. Izmaylov, J. L. Sonnenberg, Williams, F. Ding, F. Lipparini, F. Egidi, J. Goings, B. Peng, A. Petrone, T. Henderson, D. Ranasinghe, V. G. Zakrzewski, J. Gao, N. Rega, G. Zheng, W. Liang, M. Hada, M. Ehara, K. Toyota, R. Fukuda, J. Hasegawa, M. Ishida, T. Nakajima, Y. Honda, O. Kitao, H. Nakai, T. Vreven, K. Throssell, Jr. Montgomery, J. A., J. E. Peralta, F. Ogliaro, M. J. Bearpark, J. J. Heyd, E. N. Brothers, K. N. Kudin, V. N. Staroverov, T. A. Keith, R. Kobayashi, J. Normand, K. Raghavachari, A. P. Rendell, J. C. Burant, S. S. Iyengar, J. Tomasi, M. Cossi, J. M. Millam, M. Klene, C. Adamo, R. Cammi, J. W. Ochterski, R. L. Martin, K. Morokuma, O. Farkas, J. B. Foresman, D. J. Fox, Gaussian 16, Revision D. 03, Gaussian Inc.: Wallingford, CT, 2016

S8. T. Lu, F. Chen, *J. Comput. Chem.* **33**, 580–592 (2012).

S9. J. Zhang, T. Lu, *Phys. Chem. Chem. Phys.* **23**, 20323–20328 (2021).

S10. Tomasi, J.; Mennucci, B.; Cammi, R., Quantum Mechanical Continuum Solvation Models. *Chem. Rev.* **105**, 2999-3094 (2005).
